# Heterologous vector versus homologous mRNA COVID-19 booster vaccination in non-seroconverted immunosuppressed patients: a randomized controlled trial

**DOI:** 10.1038/s41467-022-33036-y

**Published:** 2022-09-12

**Authors:** Daniel Mrak, Daniela Sieghart, Elisabeth Simader, Selma Tobudic, Helga Radner, Peter Mandl, Lisa Göschl, Maximilian Koblischke, Nikolaus Hommer, Angelika Wagner, Margareta Mayer, Lorenz Schubert, Lukas Hartl, Karin Kozbial, Philipp Hofer, Felix Kartnig, Thomas Hummel, Andreas Kerschbaumer, Thomas Deimel, Antonia Puchner, Venugopal Gudipati, Renate Thalhammer, Petra Munda, Keziban Uyanik-Ünal, Andreas Zuckermann, Gottfried Novacek, Thomas Reiberger, Erika Garner-Spitzer, Roman Reindl-Schwaighofer, Renate Kain, Stefan Winkler, Josef S. Smolen, Karin Stiasny, Gottfried F. Fischer, Thomas Perkmann, Helmuth Haslacher, Markus Zeitlinger, Ursula Wiedermann, Judith H. Aberle, Daniel Aletaha, Leonhard X. Heinz, Michael Bonelli

**Affiliations:** 1 Division of Rheumatology, Department of Internal Medicine III, Medical University of Vienna, Vienna, Austria; 2 Division of Infectious Diseases and Tropical Medicine, Department of Internal Medicine I, Medical University of Vienna, Vienna, Austria; 3 Center for Virology, Medical University of Vienna, Vienna, Austria; 4 Department of Clinical Pharmacology, Medical University of Vienna, Vienna, Austria; 5 Institute of Specific Prophylaxis and Tropical Medicine, Center of Pathophysiology, Infectiology and Immunology, Medical University Vienna, Vienna, Austria; 6 Division of Gastroenterology and Hepatology, Department of Internal Medicine III, Medical University of Vienna, Vienna, Austria; 7 Department of Pathology, Medical University of Vienna, Vienna, Austria; 8 Institute of Hygiene and Applied Immunology, Center of Pathophysiology, Infectiology and Immunology, Medical University of Vienna, Vienna, Austria; 9 Department of Laboratory Medicine, Medical University of Vienna, Vienna, Austria; 10 Department of Cardiac Surgery, Medical University of Vienna, Vienna, Austria; 11 Division of Nephrology and Dialysis, Department of Internal Medicine III, Medical University of Vienna, Vienna, Austria; 12 Department of Blood Group Serology and Transfusion Medicine, Medical University of Vienna, Vienna, Austria

**Keywords:** RNA vaccines, Clinical trials, SARS-CoV-2, Immunological disorders

## Abstract

Impaired response to COVID-19 vaccination is of particular concern in immunosuppressed patients. To determine the best vaccination strategy for this vulnerable group we performed a single center, 1:1 randomized blinded clinical trial. Patients who failed to seroconvert upon two mRNA vaccinations (BNT162b2 or mRNA-1273) are randomized to receive either a third dose of the same mRNA or the vector vaccine ChAdOx1 nCoV-19. Primary endpoint is the difference in SARS-CoV-2 spike antibody seroconversion rate between vector and mRNA vaccinated patients four weeks after the third dose. Secondary outcomes include cellular immune responses. Seroconversion rates at week four are significantly higher in the mRNA (homologous vaccination, 15/24, 63%) as compared to the vector vaccine group (heterologous vaccination, 4/22, 18%). SARS-CoV-2-specific T-cell responses are reduced but could be increased after a third dose of either vector or mRNA vaccine. In a multivariable logistic regression analysis, patient age and vaccine type are associated with seroconversion. No serious adverse event is attributed to COVID-19 booster vaccination. Efficacy and safety data underline the importance of a booster vaccination and support the use of a homologous mRNA booster vaccination in immunosuppressed patients.

Trial registration: EudraCT No.: 2021-002693-10.

## Abstract (web summary)

Optimizing COVID-19 vaccination strategies for patients under immunosuppressive medication is of high importance. In this clinical trial including non-seroconverted immunosuppressed patients, a homologous mRNA booster vaccination resulted in higher seroconversion rate than a switch to a vector-based vaccine.

## Introduction

The COVID-19 pandemic poses an unprecedented challenge to public health, and several mitigation strategies exist to combat this worldwide threat. Among such strategies, COVID-19 vaccination protects against a severe disease course and leads to accelerated viral clearance^1–3^. Various types of vaccines have been approved by the European Medicines Agency (EMA), including vector vaccines, such as ChAdOx1 nCoV-19 (Oxford-AstraZeneca) or Ad26.COV2-S (Johnson&Johnson) and mRNA vaccines, such as BNT162b2 (Pfizer–BioNTech) or mRNA-1273 (Moderna)^4–7^. Most recently, NVX-CoV2373 (Novavax) and VLA2001 (Valneva) have been approved by the EMA as a protein subunit vaccine and an inactivated whole-virus COVID-19 vaccine, respectively^8,9^. Multiple elements of the innate and adaptive immune system contribute to the vaccination response^10^. One way to assess the humoral immune response to different vaccines is to measure anti-SARS-CoV-2 antibodies against the receptor-binding domain (RBD). Immunocompromised individuals are less likely to mount an adequate immune response after primary vaccination. A significant number of patients do not seroconvert upon vaccination^11–15^, leaving them more susceptible to COVID-19 infections and subsequent severe disease courses^16^. Low antibody response rates have been observed in patients with immune-mediated inflammatory diseases, haemato-oncological malignancies, those following solid-organ transplantation, and patients undergoing hemodialysis^17–21^. Several studies have reported on the efficacy and safety of an additional booster vaccination in immunosuppressed patients. These mainly consist of the administration of a third mRNA vaccine in a homologous vaccination strategy^22–26^. Evolving evidence, however, suggests that a heterologous vaccination strategy might be more efficient in nonimmunocompromised healthy volunteers^27,28^. However, data on immunogenicity and safety of homologous versus heterologous booster vaccination strategy in patients who did not seroconvert are currently limited^29–33^. We, therefore, performed a blinded randomized controlled trial to address immunogenicity and safety of the third dose in non-seroconverted immunosuppressed patients, comparing mRNA and vector vaccines.

## Results

### Patient characteristics

**Fig. 1:**
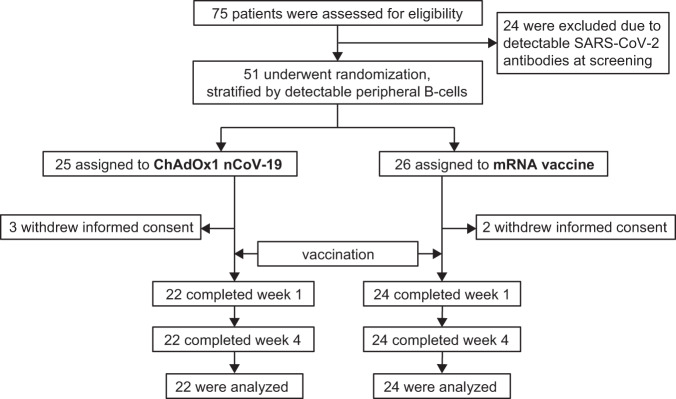
Screening, randomization, and follow-up of patients. Patients randomized to an additional mRNA vaccine dose received the same compound as with their primary vaccination. Patients were blinded to the type of vaccine used until week 4.

Seventy-five patients under immunosuppressive therapy who had been immunized with two doses of an mRNA vaccine were screened for eligibility. Twenty-four patients were excluded due to the presence of detectable SARS-CoV-2-specific antibodies. Fifty-one non-seroconverted patients were randomized, of whom 25 were assigned to receive a vector and 26 to receive an mRNA vaccine as the third dose; five patients withdrew consent between the screening and the baseline visit (Fig. 1). Thus, a total of 22/25 patients were vaccinated with a vector vaccine, and 24/26 received an mRNA vaccine. All patients subsequently presented at follow-up visits and completed the trial at week 4 after vaccination. Patient diagnoses and other characteristics were similar between the two randomized groups (Table 1).

**Table 1:** Patient characteristics at baseline

	mRNA boost	Vector boost
*n*	24	22
Age	63.4 ± 11.4	61.2 ± 14.9
Sex: female	10 (41.7)	7 (31.8)
**Diagnosis (%)**		
AIH	1 (4.2)	0 (0.0)
CTD	1 (4.2)	1 (4.5)
HTX	8 (33.3)	10 (45.5)
HTX + multiple myeloma	1 (4.2)	0 (0.0)
LiTX	2 (8.3)	3 (13.6)
LiTX + KTX	1 (4.2)	0 (0.0)
LuTX	5 (20.8)	6 (27.3)
Breast cancer	1 (4.2)	0 (0.0)
MS	2 (8.3)	0 (0.0)
Multiple myeloma	1 (4.2)	1 (4.5)
Pemphigus vulgaris	1 (4.2)	0 (0.0)
RCC	0 (0.0)	1 (4.5)
Weeks between 2nd vaccination and screening	15.2 ± 3.0	13.9 ± 4.2
Detectable peripheral B-cells (%)	23 (95.8)	21 (95.5)
Tacrolimus (%)	13 (54.2)	16 (72.7)
Mycophenolate (%)	17 (70.8)	13 (59.1)
Everolimus (%)	2 (8.3)	2 (9.1)
Sirolimus (%)	2 (8.3)	1 (4.5)
Ciclosporin (%)	2 (8.3)	2 (9.1)
Daratumumab (%)	2 (8.3)	0 (0.0)
IMiDs (%)	1 (4.2)	1 (4.5)
JAKi (%)	1 (4.2)	0 (0.0)
Hydroxychloroquine (%)	1 (4.2)	0 (0.0)
Fingolimod (%)	2 (8.3)	0 (0.0)
Vinorelbin (%)	1 (4.2)	0 (0.0)
Cabozantinib (%)	0 (0.0)	1 (4.5)
Prednisone (%)	8 (33.3)	9 (40.9)
**Number of concomitant immunosuppressants (%)**		
1	6 (25.0)	5 (22.7)
2	8 (33.3)	11 (50.0)
3	10 (41.7)	6 (27.3)
**Primary vaccination (%)**		
BNT162b2	20 (83.3)	21 (95.5)
mRNA-1273	4 (16.7)	1 (4.5)

Data are presented as *n* (%) or mean ± standard deviation (SD).

*AIH* autoimmune hepatitis, *CTD* connective tissue disease, *HTX* heart transplant, *LiTX* liver transplant, *KTX* kidney transplant, *LuTX* lung transplant, *MS* multiple sclerosis, *RCC* renal cell carcinoma, *IMiDs* immunomodulatory imide drug, *JAKi* Janus kinase inhibitor.

### Humoral vaccination response

**Fig. 2:**
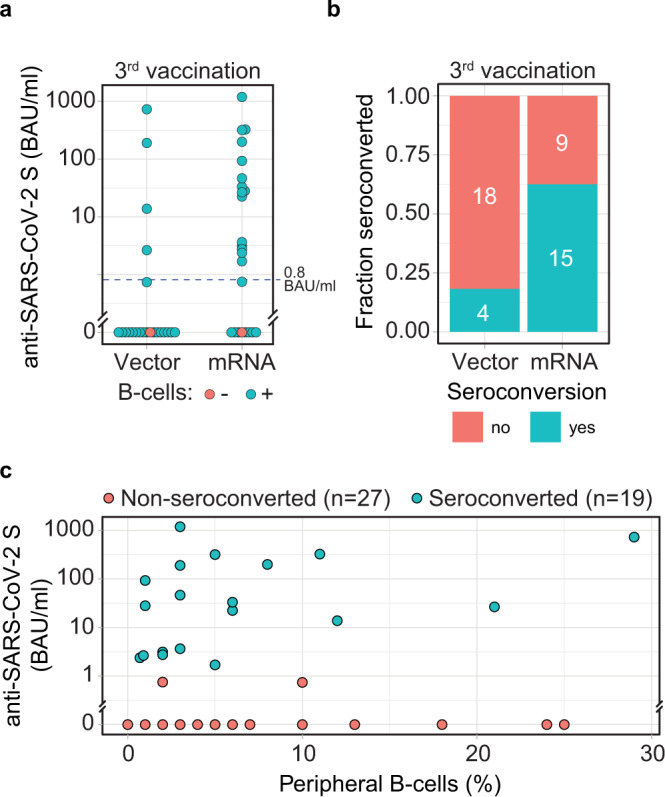
Antibody seroconversion 4 weeks after vector vs. mRNA booster vaccination. Antibodies to the receptor-binding domain (RBD) of the viral spike (S) protein were determined using an anti-SARS-CoV-2 immunoassay 4 weeks after vaccination. **a** Anti-RBD antibody levels in patients with (*n* = 44) and without (*n* = 2) peripheral B-cells, as indicated by the color of the circles. Dashed line indicates the threshold for seroconversion (0.8 BAU/ml). **b** Seroconversion rate was calculated based on the presence of anti-RBD antibodies in patients stratified by booster vaccination with vector or mRNA vaccine. **c** Anti-RBD antibody levels in patients 4 weeks after booster vaccination were correlated with levels of detectable peripheral CD19^+^ B-cells, with the color of the circles indicating status of seroconversion. Source data are provided as a Source Data file.

Seroconversion rates at week 4 after the third vaccination were significantly lower in the vector (4/22, 18%) as compared to the mRNA group (15/24, 63%; *p* = 0.006) (Fig. 2a, b). Post hoc analysis revealed that median anti-RBD antibody levels were significantly higher in mRNA- as compared to vector-vaccinated patients (*p* = 0.004, Supplementary Fig. 1a). Within mRNA vaccinees, 4/24 patients received a third dose of mRNA-1273. No significant difference was observed between BNT162b2 and mRNA-1273 (*p* = 0.253, Supplementary Fig. 1a). A more detailed patient characteristics of seroconverted and non-seroconverted patients can be found in Supplementary Table 1. In a follow-up extension study, anti-RBD antibody levels were determined 12 weeks after vaccination in 39 patients. No significant decrease in antibody levels was observed between week 4 and week 12 after the third vaccination (*p* = 0.917, Supplementary Fig. 1b).

CD19^+^ peripheral B-cells and SARS-CoV-2 spike-specific memory B-cells were analyzed by flow cytometry. Patients without detectable CD19^+^ peripheral B-cells did not develop anti-RBD antibodies in either group (Fig. 2a). No correlation was found between the numbers of peripheral B-cells and antibody levels in our patient cohort (Fig. 2c). However, SARS-CoV-2 spike-specific memory B-cells were positively associated with antibody levels in selected patients (*p* = 0.011, Supplementary Fig. 2a–c).

### Cellular vaccination response

**Fig. 3:**
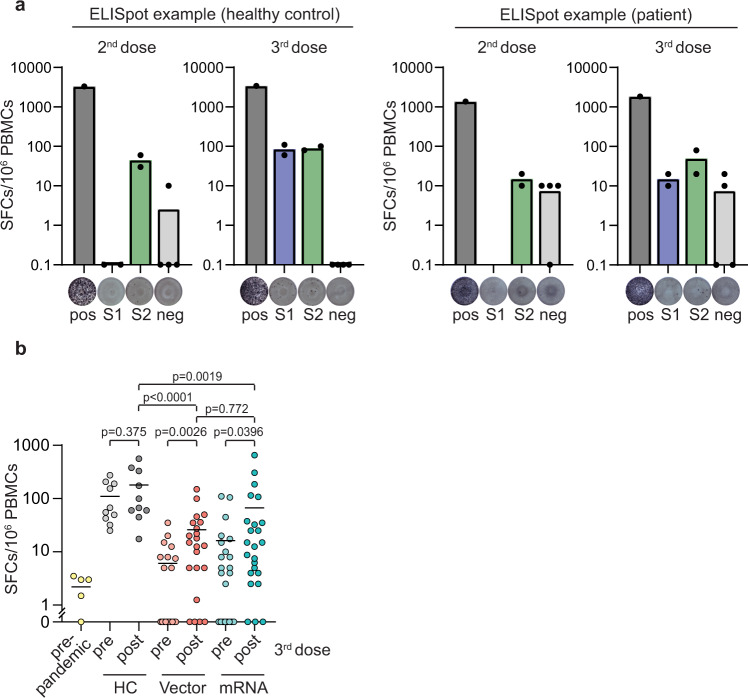
SARS-CoV-2-specific T-cell responses. **a** Representative ex vivo IFN-γ ELISpot result from peripheral blood mononuclear cells (PBMCs) stimulated or not (neg, *n* = 4) with phytohemagglutinin (PHA, *n* = 1) or spike subunit S1 and S2 peptide pools (S1, S2, *n* = 2 for each) shown for one healthy control (HC) and one patient before and after booster vaccination. Bar graphs show mean spot-forming cells (SFCs) per 10^6^ PBMCs. Dots represent individual replicates. **b** Composite ELISpot results from prepandemic controls (*n* = 5), vaccinated HC (*n* = 10), and patients before (pre, *n* = 41) and 1 week after (post, *n* = 46) third vaccination with vector and mRNA vaccine. Circles show sum of total responses from S1 and S2 peptide pools. Vertical lines indicate the mean. Paired Wilcoxon test was used to compare samples before and after booster vaccination. Mann–Whitney-U test was utilized to compare cellular vaccine responses. All tests were two-sided and no correction for multiple testing was performed. Source data are provided as a Source Data file.

SARS-CoV-2 spike-specific T-cell responses were determined by ELISpot assay before and after booster vaccination. Ten healthy controls (HC), who were vaccinated three times with BNT162b2 and developed a humoral immune response, served as a positive control. Material from patients under immunosuppressive therapy collected before the COVID-19 outbreak (*n* = 5) served as prepandemic control. Characteristics of HC and prepandemic patients can be found in Supplementary Tables 2 and 3. After primary immunization, patients under immunosuppressive therapy had reduced numbers of spike-specific T-cells as compared to vaccinated HC (Fig. 3a, b), suggesting diminished T-cell responses in immunosuppressed patients. The number of spot-forming cells (SFC) in response to the spike peptide pools (S1 and S2) significantly increased after booster vaccination with either the vector (*p* = 0.0026) or the mRNA vaccine (*p* = 0.0396). No difference in SFCs/10^6^ PBMCs was observed between patients who received the third vaccination with either an mRNA- or a vector-based vaccine (*p* = 0.772, Fig. 3a, b; Supplementary Figs. 3 and 4a, b). Similar responses were detected in individual comparisons of S1 and S2 peptide pools (Supplementary Fig. 4c). No correlation was observed between SARS-CoV-2 spike-specific T-cells and anti-RBD antibodies (Supplementary Fig. 4d). Comparable cellular immune responses against Wuhan and Omicron spike peptides were found in vector- and mRNA-vaccinated patients (Supplementary Fig. 4e).

### Predictors of vaccination response

**Fig. 4:**
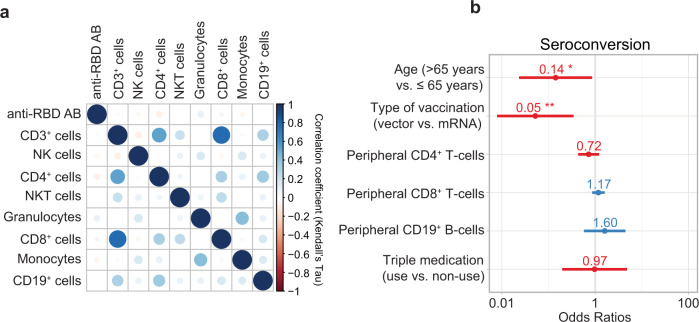
Predictors of vaccination response. **a** Pairwise correlation plot of antibodies to the receptor-binding domain (RBD) of the viral spike (S) protein after third vaccination and leukocyte subsets in patients. Correlation coefficients are displayed as Kendall’s *τ*. Source data are provided as a Source Data file. **b** Odds ratios (OR) of logistic regression assessing seroconversion after third vaccination (*n* = 46). OR were calculated using a multivariate logistic regression model. OR for peripheral CD4^+^, CD8^+^, and CD19^+^ cells are presented per 100 cells/μL. Numbers indicate OR, bars represent the 95% confidence interval. **p = 0.002, *p = 0.034.

Leukocyte subsets were analyzed before the third vaccination. No difference was observed between seroconverted and non-seroconverted patients (Supplementary Table 4). Pairwise correlations of anti-RBD antibody level at week 4 and individual cell subsets were calculated. No significant correlation was observed between anti-RBD antibody levels and any leukocyte subset (Fig. 4a and Supplementary Fig. 5), suggesting that no quantitative deficiencies contribute to seroconversion. In a multivariable logistic regression model, boost with vector vaccine and age over 65 favored the likelihood of non-seroconversion (OR 0.05 95% CI 0.01–0.28 and OR 0.14 95% CI 0.02–0.73, respectively, Fig. 4b). Accordingly, higher age was associated with lower anti-RBD antibody levels (Supplementary Fig. 6).

### Reactogenicity

Adverse events were monitored using a paper-based patient diary daily throughout the first week after vaccination and were addressed until week 4. One serious adverse event unrelated to vaccination (hospitalization for urinary tract infection) occurred during the four-week follow-up. The prevalence of systemic reactogenicity was similar in the vector and mRNA booster vaccine groups. 7/22 (32%) of vector-vaccinated patients developed arthralgia compared to 4/24 (17%) of patients with mRNA booster vaccination. Myalgia was reported in 7/22 (32%) vector-vaccinated patients compared to 9/24 (38%) mRNA-vaccinated patients. Fatigue was present in 11/22 (50%) vectors and in 10/24 (42%) mRNA-vaccinated patients. Local pain at the injection site was more frequent in mRNA (9/24, 38%) than in vector-vaccinated patients (5/22, 23%). Headache was reported in 8/24 (33%) of the mRNA-vaccinated patients compared to 9/22 (41%) of the vector-vaccinated patients.

**Fig. 5:**
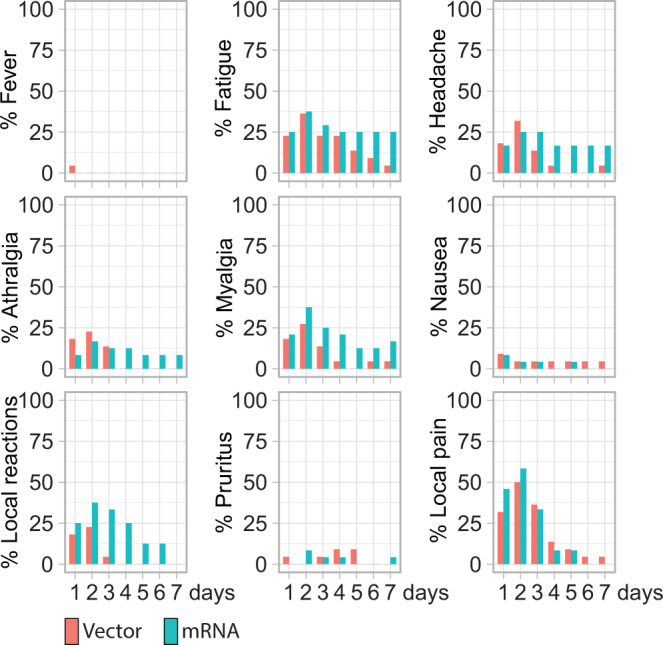
Safety. Local and systemic reactogenicity evaluated daily during the first 7 days after vaccination.

Duration of side effects was prolonged in the mRNA as compared to the vector-vaccinated group, especially for local reaction (mRNA: 1.46 versus vector 0.46 days per patient), myalgia (mRNA: 1.46, vector: 0.73 days per patient), and headache (mRNA 1.33, vector: 0.73 days per patient) (Fig. 5).

No thrombocytopenia or antibodies against platelet factor 4 (PF4) were observed after additional booster vaccination. None of the patients experienced an anaphylactoid reaction. In patients with previous organ transplantation, no acute transplant rejection was observed. Anti-HLA antibodies of patients with solid-organ transplantation were analyzed 12 weeks after the third vaccination with either a vector or an mRNA-based vaccine (*n* = 36). None of the patients developed de novo anti-HLA antibodies.

## Discussion

In this blinded randomized, controlled clinical trial, we enrolled patients under immunosuppressive therapy, who had no detectable anti-RBD antibodies upon primary vaccination with two doses of an mRNA vaccine. After booster vaccination, the seroconversion rate was significantly higher in the mRNA (homologous) than in the vector-vaccinated (heterologous) group. Overall, an additional COVID-19 vaccination resulted in humoral immune response in 41% of this initially vaccination-refractory patient population. Although the SARS-CoV-2-specific T-cell response was reduced in immunosuppressed patients as compared to HC, the cellular response could be increased after mRNA- and vector-based booster vaccination.

Patients without detectable anti-RBD antibody levels were included in our study, reflecting individuals at exceptionally high risk for severe COVID-19 disease courses. Although none of the patients seroconverted after primary vaccination, the heterogeneity of the patient cohort has to be considered since it has been reported that underlying disease-specific medication can contribute to vaccination response^34–36^. While antibody levels have been determined at multiple time points during clinical routine before booster vaccination, it cannot be ruled out that individual patients had developed transient antibodies prior to study inclusion. Although case series and clinical trials have previously addressed the immunogenicity of a third vaccination in immunosuppressed patients, data comparing homologous versus heterologous vaccination strategies are currently limited^22–26,30,33^. A significant advantage was observed for the homologous booster dose in our study: the primary outcome showed a 44% higher seroconversion rate for mRNA (homologous) versus vector (heterologous) vaccination. In line with these data, anti-RBD antibody levels were significantly higher in patients who received an mRNA-based vaccination. To assess the stability of the humoral immune response, patients were invited to participate in an open-label extension study. Anti-RBD antibody levels were sustained over a period of 12 weeks, indicating a stable humoral immune response in immunosuppressed patients over at least 3 months.

In patients under B-cell-depleting therapy, such as rituximab, we and others have previously reported that seroconversion was impaired but a cellular immune response was preserved, supporting the importance of peripheral B-cells for seroconversion^14,21,29,32,37,38^. In the current trial, we excluded patients under rituximab treatment to ensure that absence of peripheral B-cells was not the main factor for an insufficient humoral immune response to COVID-19 vaccination. In addition, patients were stratified by the presence or absence of peripheral B-cells. Overall, the numbers of CD19^+^ peripheral B-cells did not correlate with anti-RBD antibody levels. However, when we analyzed SARS-CoV-2 spike-specific memory B-cells we observed a significant correlation with anti-RBD antibody levels, suggesting that the presence of anti-SARS-CoV-2-specific memory B-cells can be interpreted as predictive of antibody production^39,40^. ELISpot assays were performed using PBMCs to determine the in vivo effect of immunosuppressants on the development of circulating spike-specific T-cells. In line with previously published data on cellular responses in patients under immunosuppressive therapy^30,35,41,42^, we observed reduced T-cell responses in our patient cohort as compared to HC, suggesting a significant contribution of impaired T-cell function to the phenomenon of non-seroconversion after primary vaccination. A significant increase in SARS-CoV-2-specific T-cells was observed for both, mRNA- and vector-based booster vaccination, which was still diminished as compared to HC. In our study, T-cell responses to spike peptide pools from Wuhan and Omicron variants showed largely comparable results. This is in line with recent data reporting that the cellular response to SARS-CoV-2 variants is preserved in most infected and vaccinated individuals^43–47^. As T-cell responses to SARS-CoV-2 spike peptides could be influenced by pre-existing cross-reactive immunity against other human corona viruses^48,49^, we analyzed responses to the more variable S1 part containing the RBD and more conserved membrane-proximal S2 moiety of the spike protein separately. The results obtained revealed some variability between S1 and S2 responses in different groups, these, however, were not statistically significant. Although our data suggest that immunosuppressive therapy leads to impaired cellular immune responses, a bigger patient cohort would be needed to define the exact role of immunosuppressants. In addition, future in vitro assays should be performed to address the specific effects of different immunosuppressants on T-cell reactivation. Furthermore, we did not observe a significant correlation between SARS-CoV-2-specific T-cells and anti-RBD antibody levels, indicating more diverse roles of immunosuppressants in antibody production.

One of the biggest concerns of an additional booster vaccination in immunosuppressed patients relates to the risk of adverse reactions, which were documented by a patient diary within our study. No serious adverse event was recorded which could be related to booster vaccination. Reactogenicity was similar upon third vaccination as reported previously in heterologous vaccination regimens ^50^. Common side effects (e.g., fatigue, headache, and myalgia) were observed, that were within the range reported in the approval studies^4–6^. While a longer duration of adverse events in patients boosted with a homologous mRNA vaccine than with the heterologous vector vaccine was observed in our patient cohort, this still lasted on average only 1.5 days.

Concerns have been raised that cell culture-derived vector vaccines can induce HLA-sensitization in transplant candidates and recipients, which may negate future transplantations or activate low-level chronic anti-graft responses^51^. To address this issue, we determined anti-HLA antibodies in solid-organ transplantation patients vaccinated either with vector or mRNA vaccine. In line with the most recent assessment report from the EMA and previous research^52,53^, no newly developed anti-HLA antibodies were detected 12 weeks post-vaccination.

One limitation of the current study is the lack of placebo control, which was considered unethical. Despite early termination due to recruitment limitations, we were still able to demonstrate the statistical significance and benefit of homologous as compared to heterologous vaccination in immunosuppressed patients, which implies a robust difference between the two currently utilized strategies. In the current study, a heterogeneous population of immunosuppressed patients was included. Therefore, additional studies with larger patient cohorts will be needed to understand the effect of disease entity and immunosuppression on seroconversion and whether the enhanced humoral and cellular immune response in initially non-seroconverted patients also translates into clinical protection. Our data show that a humoral immune response can be mounted even in non-seroconverted patients who did not respond to primary vaccination and support a homologous vaccination strategy in immunosuppressed patients, also considering the acceptable safety profile.

## Methods

### Trial design and participants

In this prospective blinded randomized controlled trial, adults (age ≥ 18 years) under immunosuppressive treatment without measurable SARS-CoV-2 spike protein-specific antibodies at least 4 weeks after their second COVID-19 vaccination were included. All patients had previously received two doses of an mRNA vaccine (BNT162b2 or mRNA-1273). Major exclusion criteria were known allergies to vaccines, previous infection with SARS-CoV-2, detectable anti-spike antibodies at the time of inclusion, or prior use of B-cell-depleting agents, such as rituximab. Healthy controls (HC) without immunosuppressive therapy as well as prepandemic immunosuppressed patients were recruited from the Vienna General Hospital and served as controls for the determination of SARS-CoV-2-specific T-cell responses. The detailed trial protocol can be found in the Supplementary Information. The trial was registered in the European Clinical Trials Database (EudraCT No.: 2021-002693-10) on the July 15, 2021.

### Randomization

The presence of detectable B-cells is critical for seroconversion^14^, therefore patients were block-randomized in a 1:1 ratio based on the presence or absence of peripheral B-cells using a computerized algorithm (Randomizer). Patients were assigned to receive either a third dose of an mRNA vaccine (BNT162b2 or mRNA-1273 containing 100 μg mRNA), corresponding to their primary vaccination compound) or a vector-based COVID-19 vaccine (ChAdOx1 nCoV-19).

### Interventions

The study was designed as described previously^29^ and is outlined in more detail in the Supplementary Information. In brief, four trial visits were performed. In addition, an optional open-label fifth visit was offered to the patients 12 weeks after vaccination. At screening, the eligibility of the patients was verified. An additional booster dose was applied at visit two (baseline), the cellular immune response was assessed at week one, the humoral immune response at week 4, and optionally at the extension visit at week 12 after the third vaccination. Safety was evaluated by a patient diary for the first week, then inquired at week 4.

### Blinding

In this trial, laboratory assessors and patients were blinded to the type of vaccine used. This was done to ensure an objective assessment of vaccine reactogenicity by the patients. Blinding of vaccines was ensured by the Central Pharmacy of the Vienna General Hospital, where dose aliquots were prearranged in syringes without reference to the vaccine type used. The study procedures followed Good Clinical Practice guidelines and the Declaration of Helsinki. The trial protocol was approved by competent authorities and the ethics committee of the Medical University of Vienna (No.: 1583/2021). At inclusion, all patients and healthy controls provided their written informed consent. All trial visits were conducted monocentric in a tertiary hospital (Vienna General Hospital). The trial started on July 22, 2021, with the inclusion of the first patient. The last patient finalized the 4-week follow-up on October 8, 2021.

### Laboratory testing

Laboratory tests, including quantification of peripheral leukocytes, anti-SARS-CoV-2 antibody testing, determination of SARS-CoV-2-specific T-and B-cell responses, and anti-HLA antibodies are described in more detail in the Supplementary Information.

### Outcomes and sample size

The primary outcome of the study was the difference in antibody seroconversion rates between the two intervention groups (vector versus mRNA vaccine). According to the manufacturer’s specification, seroconversion was defined as an anti-RBD antibody concentration of over 0.8 BAU/ml.

Secondary endpoints included overall seroconversion rate and SARS-CoV-2 antibody levels at week 4 and cellular immune response before and 1 week following vaccination. Assessment of safety included incidence and severity of adverse events over 28 days. A paper-based patient diary was used to evaluate reactogenicity. The study sample size was targeted at 150 individuals, thus based on a Chi-squared test comparing the two groups, the minimal detectable difference was 21% at a power of 80%. During recruitment, routine access to the additional vaccination dose, as offered in our trial, was facilitated for high-risk patients in Austria, which substantially slowed our inclusion rates. To allow timely study completion, the decision was taken to stop inclusion prematurely at 75 enrolled patients, which would provide power to detect an effect size of at least 32% for our patient cohort. The observed effect size was ultimately larger, which eliminates considerations about a potential type II error, i.e., failure to reject the null hypothesis although it is actually false.

### Statistical analysis

The analysis included all patients vaccinated with an additional dose. Differences in seroconversion rates were statistically compared by utilizing a Chi-squared test. Post hoc analyses, including a comparison of antibody levels, type of mRNA vaccine, antibody stability, as well cellular immunity, were performed by Kruskal–Wallis- or Mann–Whitney U test in unpaired groups or paired Wilcoxon test in paired groups. Factors associated with seroconversion rates were assessed by multivariable logistic regression analysis. Variables that might influence seroconversion were included in the model (age over 65, number of T- and B-cell subsets, type of booster vaccination, and triple immunosuppression). Univariate associations between continuous variables were described via Kendall rank correlation coefficient (*τ*). For graphical presentation, in figures with a log-scale, not-detectable anti-RBD antibodies and cellular responses have been set to 0.1. For all analyses conducted, two-sided tests were used. Data collection was facilitated by Microsoft Excel 365. GraphPad Prism (version 9.1.0) and “R” version 4.0.3 (R Development Core Team. Vienna, Austria) were used for graphical presentation and statistical analysis. Following packages were utilized: “ggplot2”, “ggbeeswarm”, “corrplot”, and “sjPlot” for creating plots, “pwr” for power calculation, and “tableone” to create baseline tables.

### Reporting summary

Further information on research design is available in the Nature Research Reporting Summary linked to this article.

## Supplementary information

Supplementary Information

Reporting Summary

## Data Availability

Source data of the main figures are provided as a source data file and in supplementary Table 5. De-identified participant data are available upon request. Proposals must be submitted to the corresponding authors and will be reviewed within 2 months. Once the proposal has been approved, data can be transferred through a secure online platform after the signing of a data access agreement and a confidentiality agreement. Source data are provided with this paper.

## Source data

Source Data

## References

[1] Pritchard E (2021). Impact of vaccination on new SARS-CoV-2 infections in the United Kingdom. Nat. Med..

[2] Dagan N (2021). BNT162b2 mRNA Covid-19 vaccine in a nationwide mass vaccination setting. N. Engl. J. Med..

[3] Singanayagam, A. et al. Community transmission and viral load kinetics of the SARS-CoV-2 delta (B.1.617.2) variant in vaccinated and unvaccinated individuals in the UK: a prospective, longitudinal, cohort study. *Lancet Infect. Dis*. **22**, 183–195 (2022). 10.1016/S1473-3099(21)00648-4 PMC8554486 34756186

[4] Voysey M (2021). Safety and efficacy of the ChAdOx1 nCoV-19 vaccine (AZD1222) against SARS-CoV-2: an interim analysis of four randomised controlled trials in Brazil, South Africa, and the UK. Lancet Lond. Engl..

[5] Polack FP (2020). Safety and efficacy of the BNT162b2 mRNA Covid-19 vaccine. N. Engl. J. Med..

[6] Baden LR (2021). Efficacy and safety of the mRNA-1273 SARS-CoV-2 vaccine. N. Engl. J. Med..

[7] Sadoff J (2021). Safety and efficacy of single-dose Ad26.COV2.S vaccine against Covid-19. N. Engl. J. Med..

[8] Heath PT (2021). Safety and efficacy of NVX-CoV2373 Covid-19 vaccine. N. Engl. J. Med..

[9] EMA. *COVID-19 Vaccine (inactivated, adjuvanted) Valneva. European Medicines Agency.* https://www.ema.europa.eu/en/medicines/human/EPAR/covid-19-vaccine-inactivated-adjuvanted-valneva. (2022).

[10] Sette A, Crotty S (2021). Adaptive immunity to SARS-CoV-2 and COVID-19. Cell.

[11] Stampfer SD (2021). Response to mRNA vaccination for COVID-19 among patients with multiple myeloma. Leukemia.

[12] Grupper A (2021). Reduced humoral response to mRNA SARS-CoV-2 BNT162b2 vaccine in kidney transplant recipients without prior exposure to the virus. Am. J. Transplant..

[13] Boyarsky BJ (2021). Antibody response to 2-dose SARS-CoV-2 mRNA vaccine series in solid organ transplant recipients. JAMA.

[14] Mrak D (2021). SARS-CoV-2 vaccination in rituximab-treated patients: B cells promote humoral immune responses in the presence of T-cell-mediated immunity. Ann. Rheum. Dis..

[15] Bonelli, M. M., Mrak, D., Perkmann, T., Haslacher, H. & Aletaha, D. SARS-CoV-2 vaccination in rituximab-treated patients: evidence for impaired humoral but inducible cellular immune response. *Ann. Rheum. Dis*. 10.1136/annrheumdis-2021-220408 (2021). 10.1136/annrheumdis-2021-220408 33958323

[16] Qin CX (2021). Risk of breakthrough SARS-CoV-2 infections in adult transplant recipients. Transplantation.

[17] Simon B (2021). Haemodialysis patients show a highly diminished antibody response after COVID-19 mRNA vaccination compared with healthy controls. Nephrol. Dial. Transpl..

[18] Mair, M. J. et al. Humoral immune response in hematooncological patients and health care workers who received SARS-CoV-2 vaccinations. *JAMA Oncol*. 10.1001/jamaoncol.2021.5437 (2021). 10.1001/jamaoncol.2021.5437 PMC8485209 34591965

[19] Picchianti-Diamanti, A. et al. Immunosuppressive therapies differently modulate humoral- and T-cell-specific responses to COVID-19 mRNA vaccine in rheumatoid arthritis patients. *Front. Immunol*. **12**, 10.3389/fimmu.2021.740249 (2021). 10.3389/fimmu.2021.740249 PMC8477040 34594343

[20] Farroni, C. et al. Kinetics of the B- and T-cell immune responses after 6 months from SARS-CoV-2 mRNA vaccination in patients with rheumatoid arthritis. *Front. Immunol*. **13**, 10.3389/fimmu.2022.846753 (2022). 10.3389/fimmu.2022.846753 PMC8924958 35309297

[21] Tortorella C (2022). Humoral- and T-cell–specific immune responses to SARS-CoV-2 mRNA vaccination in patients with MS using different disease-modifying therapies. Neurology.

[22] Hall, V. G. et al. Randomized trial of a third dose of mRNA-1273 vaccine in transplant recipients. *N. Engl. J. Med*. 10.1056/NEJMc2111462 (2021). 10.1056/NEJMc2111462 PMC8385563 34379917

[23] Werbel, W. A. et al. Safety and immunogenicity of a third dose of SARS-CoV-2 vaccine in solid organ transplant recipients: a case series. *Ann. Intern. Med*. 10.7326/L21-0282 (2021). 10.7326/L21-0282 PMC8252023 34125572

[24] Connolly, C. M. et al. Booster-dose SARS-CoV-2 vaccination in patients with autoimmune disease: a case series. *Ann. Rheum. Dis*. 10.1136/annrheumdis-2021-221206 (2021). 10.1136/annrheumdis-2021-221206 PMC11034903 34493492

[25] Westhoff, T. H. et al. A third vaccine dose substantially improves humoral and cellular SARS-CoV-2 immunity in renal transplant recipients with primary humoral non-response. *Kidney Int*. 10.1016/j.kint.2021.09.001 (2021). 10.1016/j.kint.2021.09.001 PMC8427909 34509489

[26] Kamar N (2021). Three doses of an mRNA Covid-19 vaccine in solid-organ transplant recipients. N. Engl. J. Med..

[27] Munro, A. P. S. et al. Safety and immunogenicity of seven COVID-19 vaccines as a third dose (booster) following two doses of ChAdOx1 nCov-19 or BNT162b2 in the UK (COV-BOOST): a blinded, multicentre, randomised, controlled, phase 2 trial. *Lancet* **398**, 2258–2276 (2021). 10.1016/S0140-6736(21)02717-3 PMC8639161 34863358

[28] Atmar RL (2022). Homologous and heterologous Covid-19 booster vaccinations. N. Engl. J. Med..

[29] Bonelli, M. et al. Additional heterologous versus homologous booster vaccination in immunosuppressed patients without SARS-CoV-2 antibody seroconversion after primary mRNA vaccination: a randomised controlled trial. *Ann. Rheum. Dis*. 10.1136/annrheumdis-2021-221558 (2022). 10.1136/annrheumdis-2021-221558 35027397

[30] Schrezenmeier E (2021). B and T cell responses after a third dose of SARS-CoV-2 vaccine in kidney transplant recipients. J. Am. Soc. Nephrol..

[31] Lyski, Z. L. et al. *Immunogenicity of Pfizer mRNA COVID-19 vaccination followed by J&J adenovirus COVID-19 vaccination in two CLL patients*. 10.1101/2021.09.02.21262146. (2021). 10.1155/2022/6831640 PMC8815431 35127183

[32] Simon, D. et al. Efficacy and safety of SARS-CoV-2 revaccination in non-responders with immune-mediated inflammatory disease. *Ann. Rheum. Dis*. 10.1136/annrheumdis-2021-221554 (2021). 10.1136/annrheumdis-2021-221554 34819271

[33] Reindl-Schwaighofer, R. et al. Comparison of SARS-CoV-2 antibody response 4 weeks after homologous vs heterologous third vaccine dose in kidney transplant recipients: a randomized clinical trial. *JAMA Intern. Med*. 10.1001/jamainternmed.2021.7372 (2021). 10.1001/jamainternmed.2021.7372 PMC8689434 34928302

[34] Broseta JJ (2021). Humoral and cellular responses to mRNA-1273 and BNT162b2 SARS-CoV-2 vaccines administered to hemodialysis patients. Am. J. Kidney Dis..

[35] Cucchiari D (2021). Cellular and humoral response after MRNA-1273 SARS-CoV-2 vaccine in kidney transplant recipients. Am. J. Transplant..

[36] Rodríguez-Espinosa D (2022). Incidence of severe breakthrough SARS-CoV-2 infections in vaccinated kidney transplant and haemodialysis patients. J. Nephrol..

[37] Moor, M. B. et al. Humoral and cellular responses to mRNA vaccines against SARS-CoV-2 in patients with a history of CD20 B-cell-depleting therapy (RituxiVac): an investigator-initiated, single-centre, open-label study. *Lancet Rheumatol*. 10.1016/S2665-9913(21)00251-4 (2021). 10.1016/S2665-9913(21)00251-4 PMC8423431 34514436

[38] D’Abramo A (2021). Prolonged and severe SARS-CoV-2 infection in patients under B-cell-depleting drug successfully treated: a tailored approach. Int. J. Infect. Dis..

[39] Goel RR (2021). Distinct antibody and memory B cell responses in SARS-CoV-2 naïve and recovered individuals after mRNA vaccination. Sci. Immunol..

[40] Ciabattini, A. et al. Evidence of SARS-CoV-2-specific memory B cells six months after vaccination with the BNT162b2 mRNA vaccine. *Front. Immunol*. 10.3389/fimmu.2021.740708 (2021). 10.3389/fimmu.2021.740708 PMC8505800 34650563

[41] Schmidt T (2021). Cellular immunity predominates over humoral immunity after homologous and heterologous mRNA and vector-based COVID-19 vaccine regimens in solid organ transplant recipients. Am. J. Transplant..

[42] Schramm R (2021). Poor humoral and T-cell response to two-dose SARS-CoV-2 messenger RNA vaccine BNT162b2 in cardiothoracic transplant recipients. Clin. Res. Cardiol..

[43] De Marco L (2022). Assessment of T-cell reactivity to the SARS-CoV-2 Omicron variant by immunized individuals. JAMA Netw. Open.

[44] Naranbhai V (2022). T cell reactivity to the SARS-CoV-2 Omicron variant is preserved in most but not all individuals. Cell.

[45] Mazzoni, A. et al. SARS-CoV-2 spike-specific CD4+ T cell response is conserved against variants of concern, including Omicron. *Front. Immunol*. **13**, 10.3389/fimmu.2022.801431 (2022). 10.3389/fimmu.2022.801431 PMC8826050 35154116

[46] Geers D (2021). SARS-CoV-2 variants of concern partially escape humoral but not T cell responses in COVID-19 convalescent donors and vaccine recipients. Sci. Immunol..

[47] Keeton R (2022). T cell responses to SARS-CoV-2 spike cross-recognize Omicron. Nature.

[48] Grifoni A (2020). Targets of T cell responses to SARS-CoV-2 Coronavirus in humans with COVID-19 disease and unexposed individuals. Cell.

[49] Mateus J (2020). Selective and cross-reactive SARS-CoV-2 T cell epitopes in unexposed humans. Science.

[50] Borobia AM (2021). Immunogenicity and reactogenicity of BNT162b2 booster in ChAdOx1-S-primed participants (CombiVacS): a multicentre, open-label, randomised, controlled, phase 2 trial. Lancet.

[51] Abu-Khader A (2022). SARS Cov-2 vaccination induces de novo donor-specific HLA antibodies in a renal transplant patient on waiting list: a case report. HLA.

[52] Russo G (2022). SARS-COV-2 vaccination with BNT162B2 in renal transplant patients: Risk factors for impaired response and immunological implications. Clin. Transplant..

[53] EMA: Committee for Medicinal Products for Human Use (CHMP). *Assessment report: COVID-19 Vaccine AstraZeneca*. https://www.ema.europa.eu/en/documents/assessmentreport/vaxzevria-previously-covid-19-vaccine-astrazeneca-epar-public-assessment-report_en.pdf (2021).

