# Supplementary material for: Heterologous vector versus homologous mRNA COVID-19 booster vaccination in non-seroconverted immunosuppressed patients: a randomized controlled trial
Source: Nat Commun. 2022 Sep 12;13:5362. doi: 10.1038/s41467-022-33036-y (PMC9467419; doi:10.1038/s41467-022-33036-y)
Supplement: Supplementary file 1 — Supplementary Information [file 41467_2022_33036_MOESM1_ESM.pdf]

Supplementary Information

Heterologous vector versus homologous mRNA COVID-19 booster vaccination in non-seroconverted immunosuppressed patients: a randomized controlled trial

Daniel Mrak, Daniela Sieghart, Elisabeth Simader, Selma Tobudic, Helga Radner, Peter Mandl, Lisa Göschl, Maximilian Koblischke, Nikolaus Hommer, Angelika Wagner, Margareta Mayer, Lorenz Schubert, Lukas Hartl, Karin Kozbial, Philipp Hofer, Felix Kartnig, Thomas Hummel, Andreas Kerschbaumer, Thomas Deimel, Antonia Puchner, Venugopal Gudipati, Renate Thalhammer, Petra Munda, Keziban Uyanik-Ünal, Andreas Zuckermann, Gottfried Novacek, Thomas Reiberger, Erika Garner-Spitzer, Roman Reindl-Schwaighofer, Renate Kain, Stefan Winkler, Josef S. Smolen, Karin Stiasny, Gottfried F. Fischer, Thomas Perkmann, Helmuth Haslacher, Markus Zeitlinger, Ursula Wiedermann, Judith H. Aberle, Daniel Aletaha, Leonhard X. Heinz and Michael Bonelli

Supplementary Figure 1

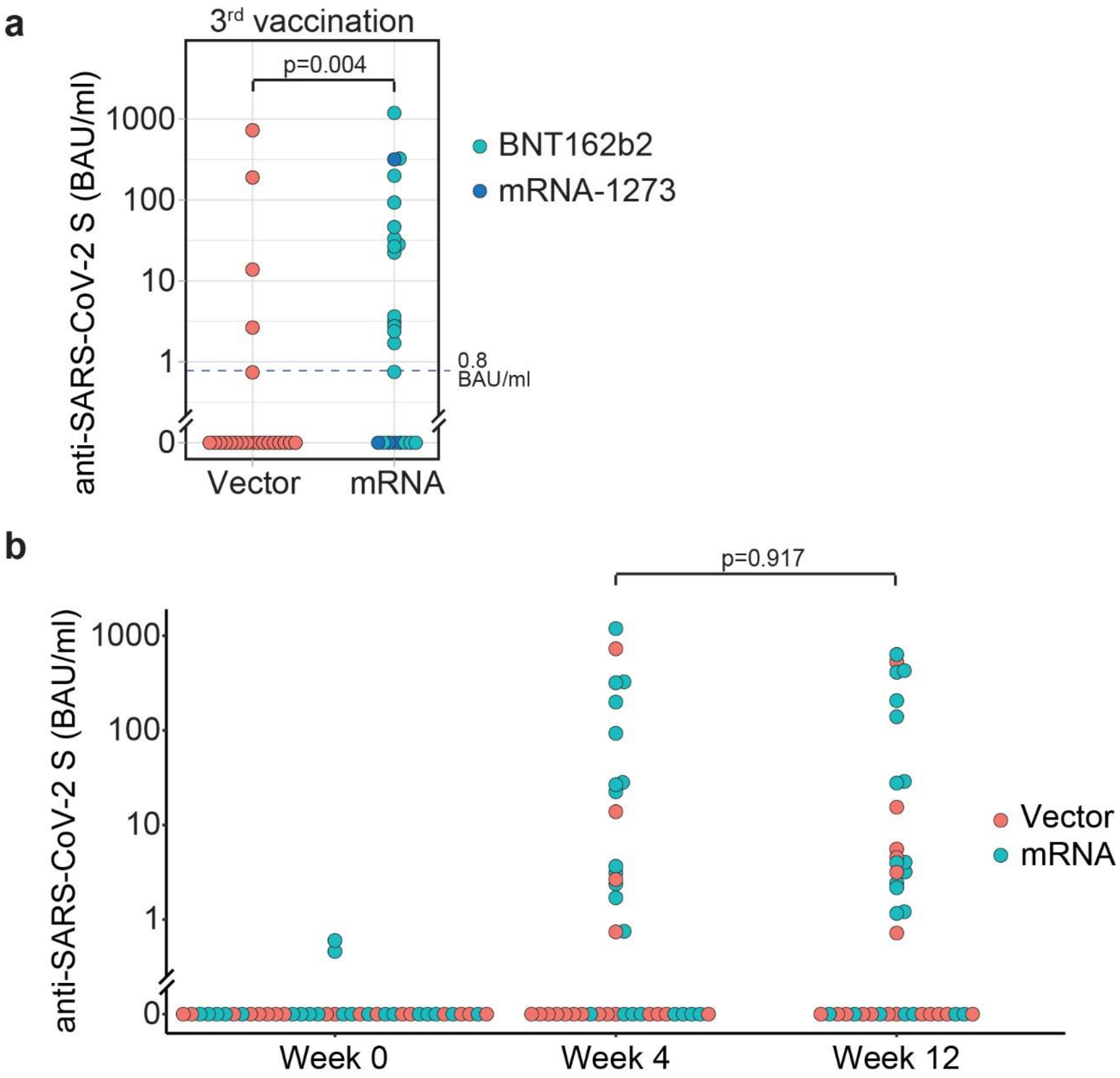

Supplementary Figure 1. Related to Figure 2. **a.** Anti-RBD antibody levels in patients four weeks after third vaccination as indicated. Dashed line indicates threshold for seroconversion (0.8 BAU/ml). Two sided Kruskal-Wallis test was performed to compare antibody levels between the groups. **b.** Anti-RBD antibody levels in patients before, 4 and 12 weeks after the third vaccination as indicated. Paired Wilcoxon test was used to compare antibody levels at week 4 and 12.

Supplementary Figure 2

a

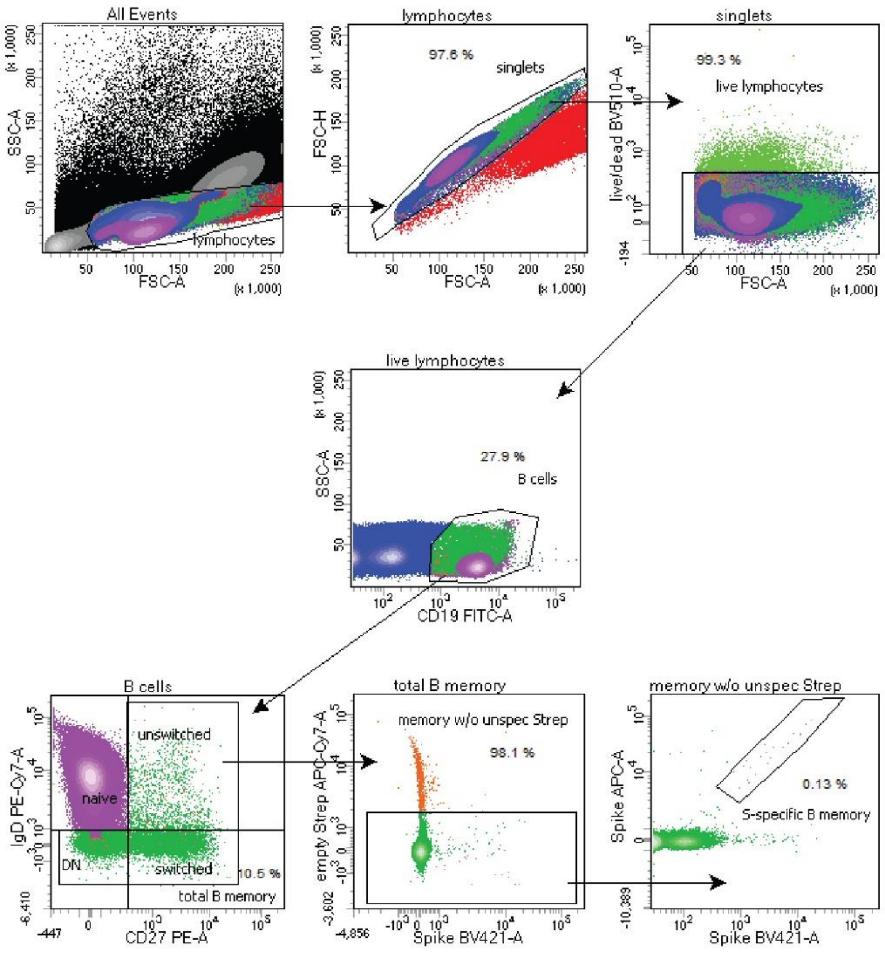

b

c

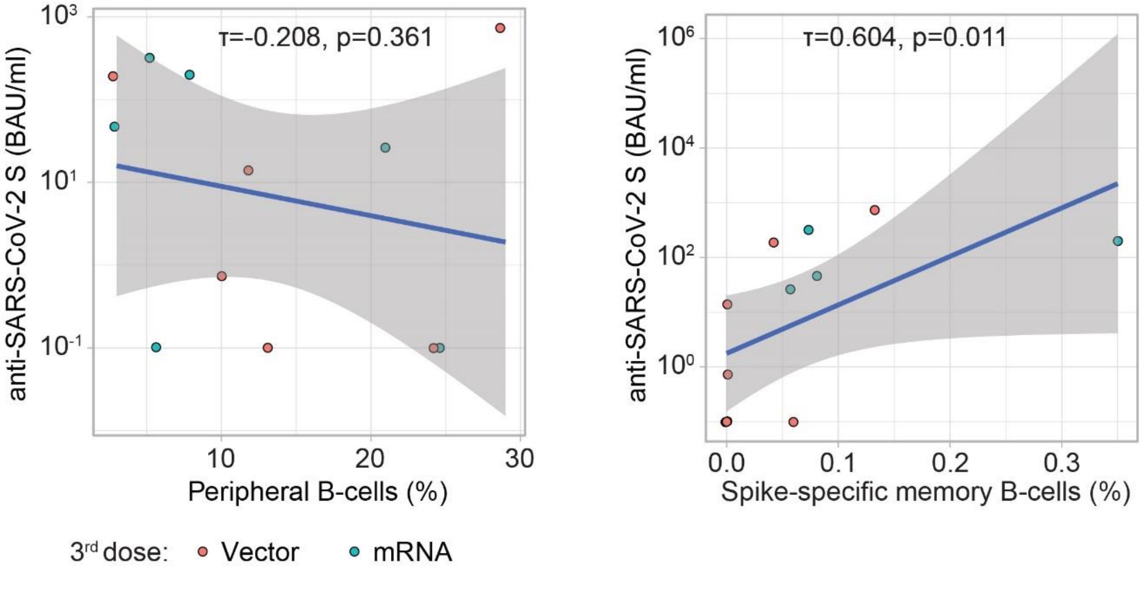

Supplementary Figure 2. Related to Figure 2. **a.** Gating strategy for quantification of SARS-CoV-2 spike-specific memory B-cells. FSC/SSC was used to select lymphocytes and remove doublets. Dead cells were excluded by using fixable viability dye eFluor-506. $CD19^+$ B-cells were gated and S-specific memory B-cells were quantified as percentages of total memory B-cells, including the $CD19^+$ un-switched ($IgD^+/CD27^+$), switched (IgD-/$CD27^+$) and double-negative ($IgD^-/CD27^-$) memory subset after exclusion of cells positive for streptavidin-APC-Cy7 without biotinylated protein that was used as a decoy probe to gate out B cells that unspecifically bind streptavidin. **b,c.** Anti-RBD antibodies in relation with relative detectable peripheral B-cells **(b)** and with relative SARS-CoV-2 spike-specific memory B-cells **(c)** in 12 selected patients. Color indicates type of vaccine used. Grey area represents the 95% confidence interval.

Supplementary Figure 3

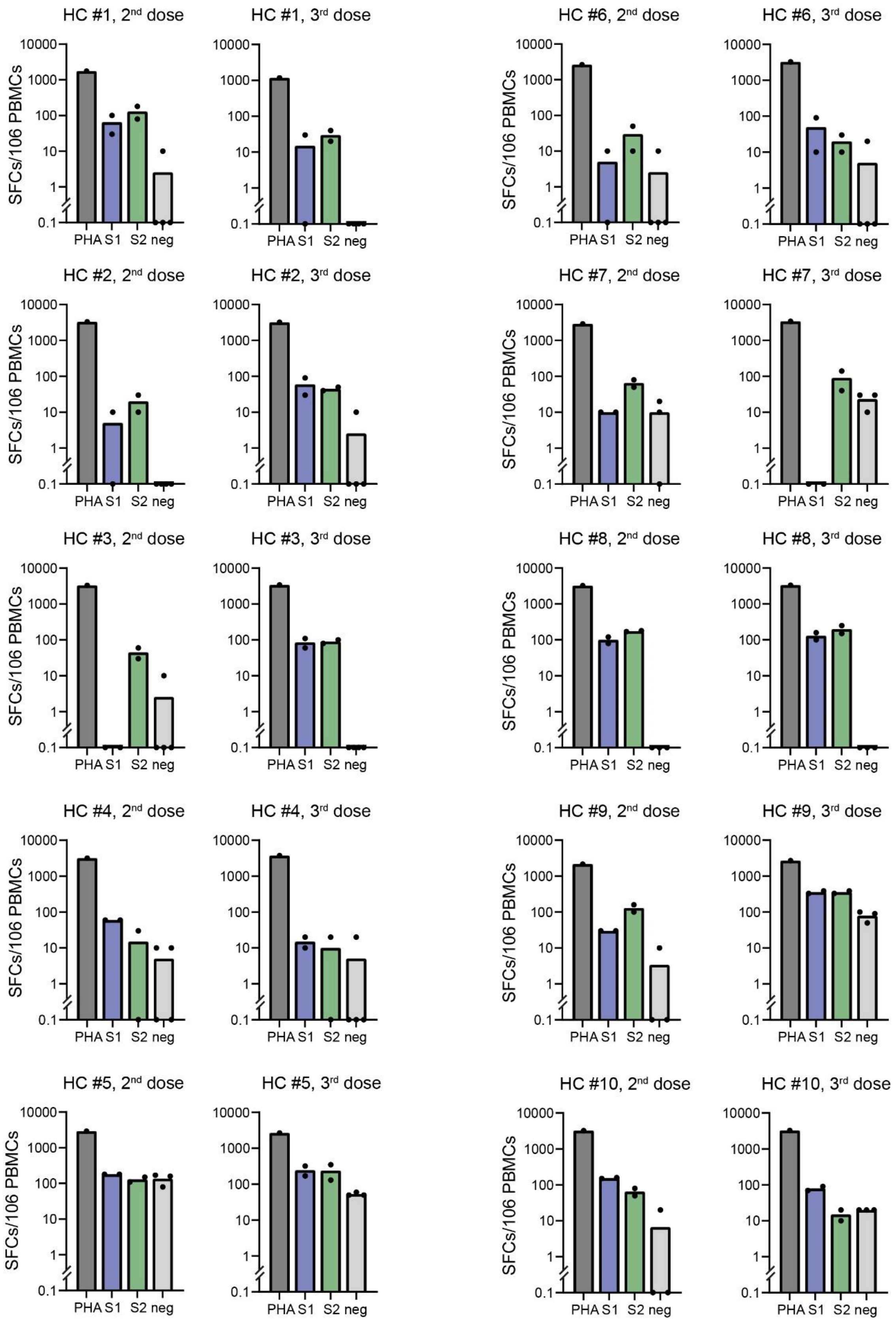

Supplementary Figure 3. Related to Figure 3. E*x vivo* IFN-γ ELISpot result from peripheral blood mononuclear cells (PBMCs) stimulated or not (neg) with phytohemagglutinin (PHA) or spike subunit S1 and S2 peptide pools (S1, S2) shown for all healthy controls (n=10) before and one week after booster vaccination. Bar graphs show mean spot forming cells (SFCs) per 10^6 PBMCs. Dots represent individual replicates.

Supplementary Figure 4

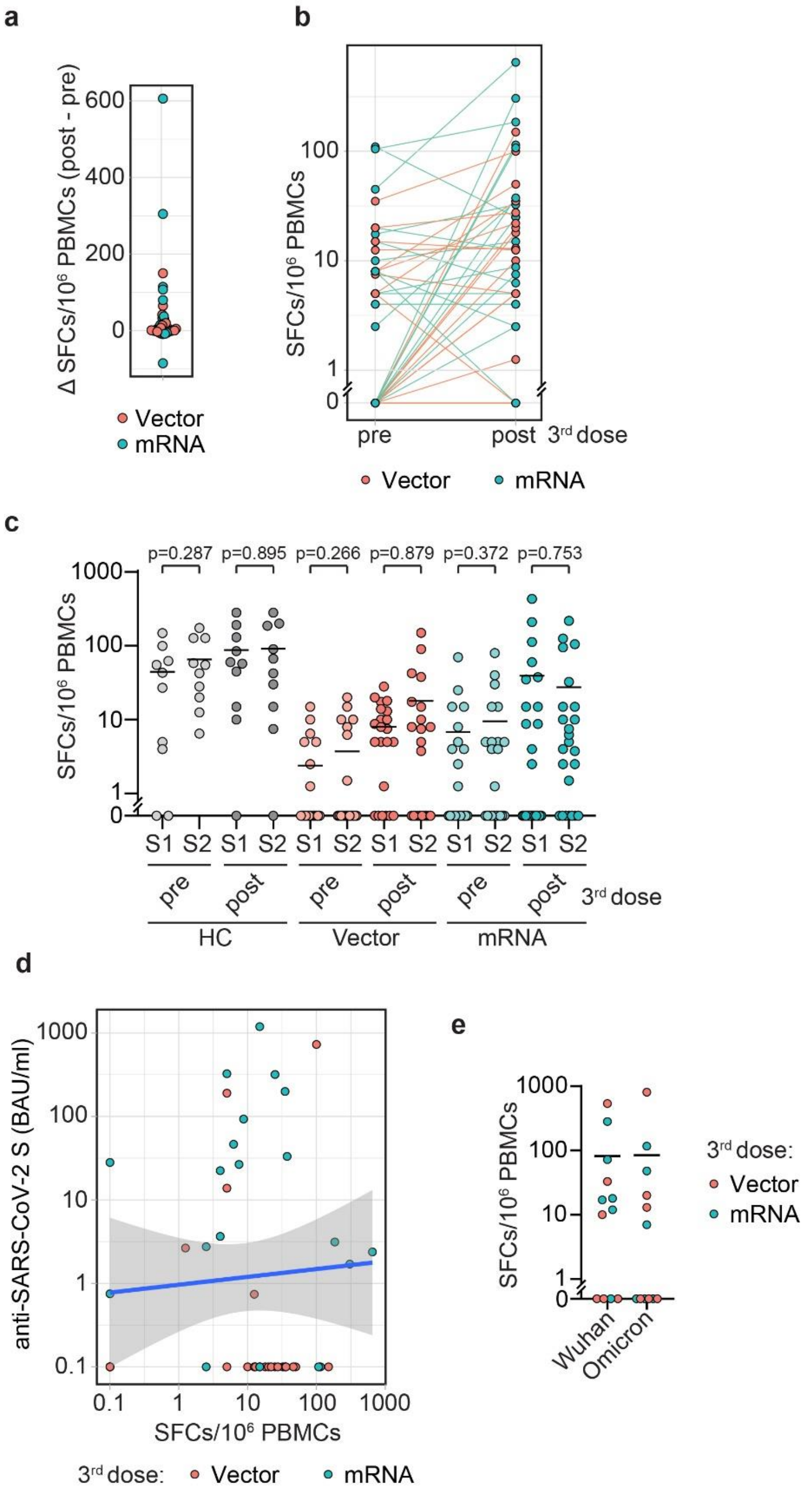

Supplementary Figure 4. a,b. Related to Figure 3. E*x vivo* IFN-γ ELISpot results from patients indicating change in SFCs/10^6 PBMCs before (pre) and one week after (post) third vaccination. **a.** Difference (post – pre) and **b.** individual SFCs/10^6 PBMCs before (pre) and one week after (post) third vaccination from patients as indicated. **c.** Composite ELISpot results from vaccinated HCs (n=10) and patients before (pre, n=41) and one week after (post, n=46) third vaccination with vector and mRNA vaccine. Circles show individual responses from S1 and S2 peptide pools as indicated. Vertical line indicates mean. Two-sided Wilcoxon test was performed to compare S1 and S2 peptide pools. **d.** Anti-RBD antibody levels in patients four weeks after booster vaccination correlated with e*x vivo* IFN-γ ELISpot results one week after booster vaccination as indicated. Grey area represents the 95% confidence interval. **e.** Composite Wuhan and Omicron-specific IFN-γ ELISpot results from patients (n=12) after third vaccination with vector (n=6) and mRNA vaccine (n=6). Circles show sum of total responses from S1 and S2 peptide pools. Vertical line indicates mean.

Supplementary Figure 5

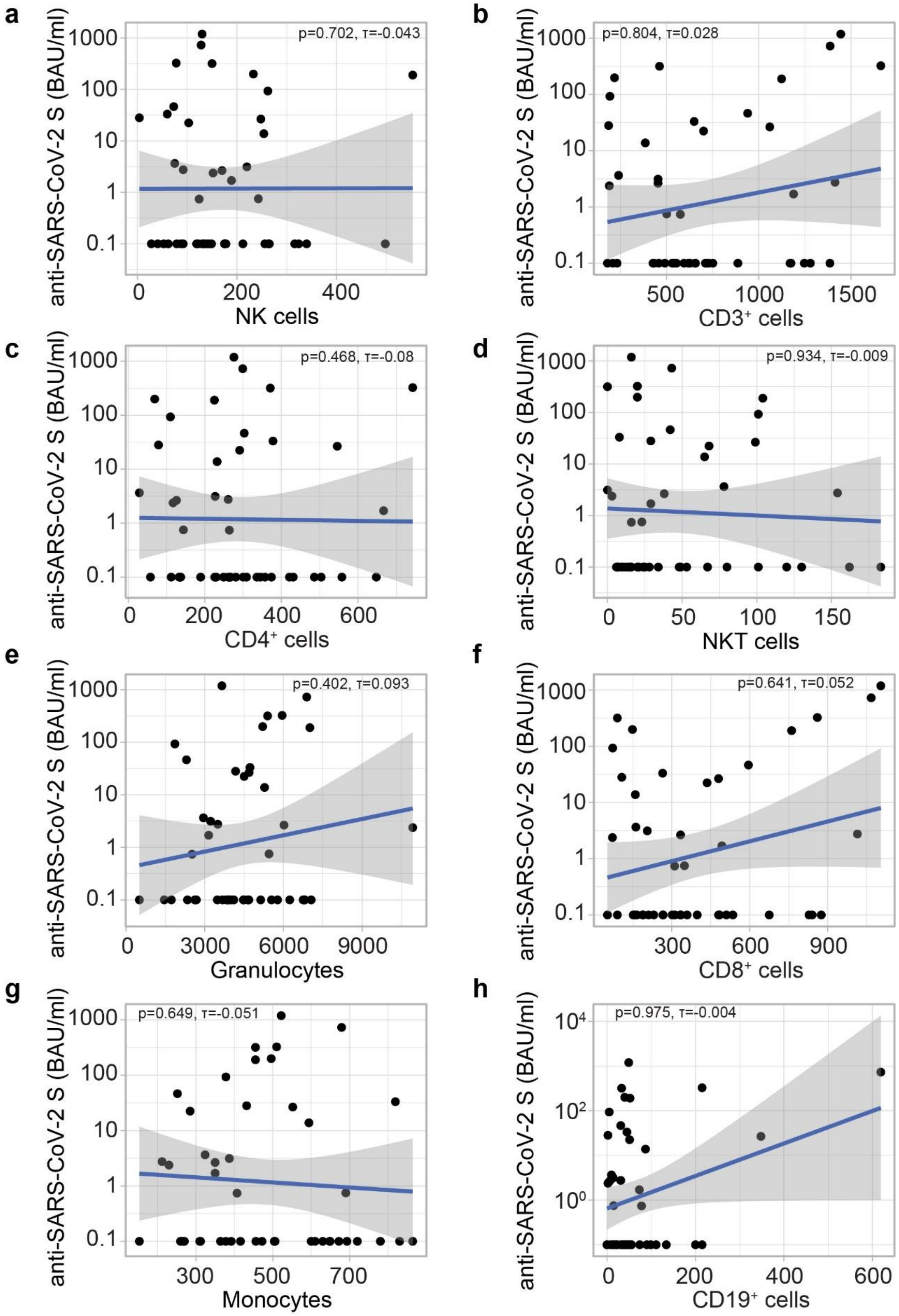

Supplementary Figure 5. Related to Figure 4. Anti-RBD antibody levels in patients four weeks after booster vaccination correlated with leukocyte subsets (cells/µl), specifically **(a)** NK cells **(b)** $CD3^+$ cells **(c)** $CD4^+$ cells **(d)** NKT cells **(e)** Granulocytes **(f)** $CD8^+$ cells **(g)** Monocytes **(h)** $CD19^+$ cells analyzed before vaccination as indicated. Correlations were calculated using the Kendall rank correlation coefficient, grey area represents the 95% confidence interval.

Supplementary Figure 6

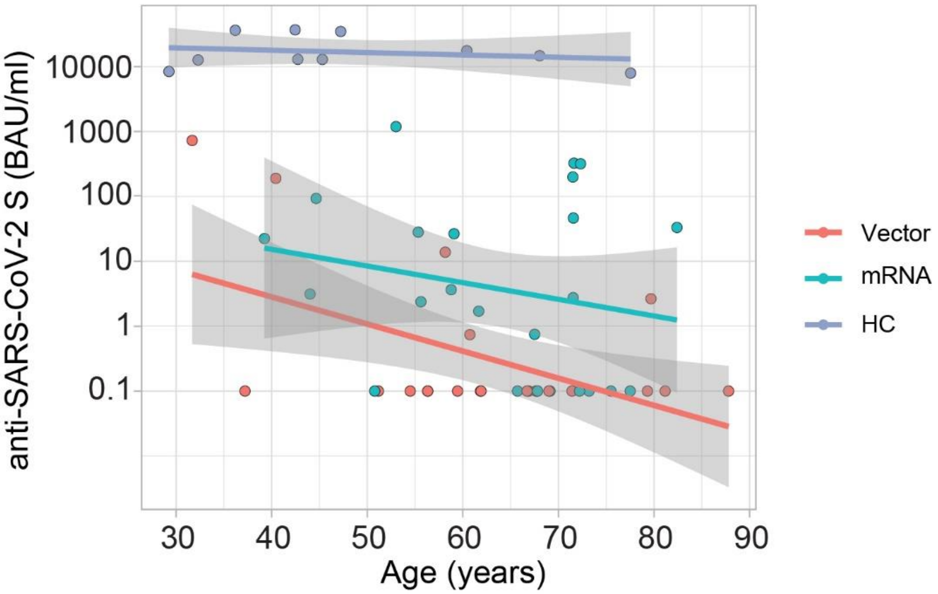

Supplementary Figure 6. Related to Figure 4. Anti-RBD antibody levels in boosted healthy controls (n=10) as well as vector (n=22) or mRNA (n=24) vaccinated patients were correlated with age. Grey area represents the 95% confidence interval.

Supplementary Table 1

Seroconversion	no	yes
n	27	19
Age	65.3 ± 10.6	58.1 ± 15.3
Sex: female	6 (22.2)	11 (57.9)
Diagnosis (%)		
AIH	1 (3.7)	0 (0.0)
CTD	0 (0.0)	2 (10.5)
HTX	14 (51.9)	4 (21.1)
HTX + Multiple myeloma	1 (3.7)	0 (0.0)
LiTX	4 (14.8)	1 (5.3)
LiTX+KTX	1 (3.7)	0 (0.0)
LuTX	5 (18.5)	6 (31.6)
Breast cancer	0 (0.0)	1 (5.3)
MS	0 (0.0)	2 (10.5)
Multiple myeloma	1 (3.7)	1 (5.3)
Pemphigus vulgaris	0 (0.0)	1 (5.3)
RCC	0 (0.0)	1 (5.3)
Time between 2nd vaccination and screening	14.35 ± 3.73	14.83 ± 3.54
Detectable peripheral B-cells (%)	25 (92.6)	19 (100.0)
Tacrolimus (%)	19 (70.4)	10 (52.6)
Mycophenolate (%)	19 (70.4)	11 (57.9)
Everolimus (%)	3 (11.1)	1 (5.3)
Sirolimus (%)	2 (7.4)	1 (5.3)
Ciclosporin (%)	3 (11.1)	1 (5.3)
Daratumumab (%)	1 (3.7)	1 (5.3)
IMiDs (%)	1 (3.7)	1 (5.3)
JAKi (%)	1 (3.7)	0 (0.0)
Hydroxychloroquine (%)	0 (0.0)	1 (5.3)
Fingolimod (%)	0 (0.0)	2 (10.5)
Vinorelbin (%)	0 (0.0)	1 (5.3)
Cabozantinib (%)	0 (0.0)	1 (5.3)
Prednisone (%)	9 (33.3)	8 (42.1)
Number of immunosuppressants (%)		
1	5 (18.5)	6 (31.6)
2	13 (48.1)	6 (31.6)
3	9 (33.3)	7 (36.8)
Type of booster vaccine		
ChAdOx1 nCoV-19	18 (66.7)	4 (21.1)
BNT162b2	6 (22.2)	14 (73.7)

mRNA-1273	3 (11.1)	1 (5.3)
SARS-CoV-2-S Protein antibodies in BAU/ml (median [IQR])	0.0 [0.0, 0.0]	28.1 [3.4, 194.5]

Data are presented as n (%), mean ± standard deviation (SD) or as median with interquartile range (IQR), AIH: Autoimmune hepatitis, CTD: Connective tissue disease, HTX: Heart transplant, LiTX: Liver transplant, KTX: Kidney transplant, LuTX: Lung transplant, MS: Multiple sclerosis, RCC: Renal cell carcinoma, IMiDs: Immunomodulatory imide drug, JAKi: Janus kinase inhibitor

Supplementary Table 2

Healthy controls	
n	10
Age (years)	48.2 (± 15.8)
Sex: female (%)	4 (40.0)
Vaccine: BNT162b2 (%)	10 (100.0)
SARS-CoV-2 spike AB (median [IQR])	13850 [12732, 30502]

Baseline characteristics of healthy controls, all vaccinated three times with BNT162b2, SARS-CoV-2 spike antibodies (AB) (BAU/ml) were determined 4 weeks after the third dose.

Supplementary Table 3

n	5
Age (years)	54.6 (± 13.7)
Sex: female (%)	4 (80.0%)
Diagnosis (%)	
KTX	5 (100.0)
Immunosuppressive Treatment	
Tacrolimus	5 (100.0)
Mycophenolate	5 (100.0)
Prednisone	5 (100.0)

Baseline characteristics of pre-pandemic immunosuppressed controls
KTX: kidney transplantation

Supplementary Table 4

Seroconversion	no	yes
n	27	19
leukocytes (mean (SD))	5670.37 (1942.60)	6268.42 (2227.87)
CD4-CD8 cell ratio (mean (SD))	1.03 (0.66)	0.98 (0.90)
$CD3^+$ cells (mean (SD))	679.26 (323.21)	755.47 (500.81)
NK cells (mean (SD))	175.85 (108.89)	167.05 (119.30)
$CD4^+$ cells (mean (SD))	297.04 (143.89)	281.53 (195.70)
NKT cells (mean (SD))	47.41 (49.95)	48.26 (42.87)
Granulocytes (mean (SD))	4243.30 (1744.75)	4811.26 (2072.02)
$CD8^+$ cells (mean (SD))	366.63 (224.44)	443.68 (358.26)
Monocytes (mean (SD))	512.96 (193.28)	435.89 (157.01)
$CD19^+$ cells (mean (SD))	58.52 (54.55)	90.16 (153.42)

Leukocyte subsets were determined by flow cytometry in seroconverted and non-seroconverted patients. Mean ± standard deviation (SD) of absolute numbers (cells/µl) are shown.

Supplementary Table 5

Patient	Immunosuppressive treatment	Type of vaccine	Anti-SARS-CoV-2 RBD antibodies (BAU/ml)	Cellular vaccine response
1	Everolimus	Vector	< 0.4	0
2	Tacrolimus, Everolimus, Daratumab	mRNA	< 0.4	0
3	IMiD, Prednisone	Vector	< 0.4	10
4	Mycophenolate	mRNA	1.7	305
5	Mycophenolate, Prednisone, Hydroxychloroquine	mRNA	3.13	185
6	Mycophenolate, Tacrolimus	Vector	728	100
7	Fingolimod	mRNA	3.65	4
8	Mycophenolate, Prednisone	mRNA	1190	15
9	IMiD, Daratumumab, Prednisone	mRNA	28.1	0
10	Tacrolimus, Everolimus	mRNA	< 0.4	2.5
11	Mycophenolate, Tacrolimus, Prednisone	Vector	< 0.4	50
12	Mycophenolate, Sirolimus, JAKi	mRNA	< 0.4	12.5
13	Mycophenolate, Tacrolimus	mRNA	< 0.4	32.5
14	Tacrolimus, Prednisone	Vector	190	5
15	Tacrolimus, Prednisone	Vector	< 0.4	5
16	Mycophenolate, Tacrolimus, Prednisone	mRNA	22.4	4
17	Mycophenolate, Tacrolimus, Prednisone	Vector	< 0.4	35
18	Mycophenolate, Tacrolimus	Vector	< 0.4	0
19	Tacrolimus, Everolimus	Vector	13.8	5
20	Mycophenolate, Ciclopsorin, Prednisone	Vector	< 0.4	0
21	Mycophenolate, Sirolimus	mRNA	33.2	37.5
22	Mycophenolate, Tacrolimus	Vector	< 0.4	150
23	Mycophenolate, Tacrolimus, Prednisone	Vector	< 0.4	18
24	Mycophenolate, Tacrolimus	mRNA	0.75	0
25	Mycophenolate, Ciclopsorin	Vector	< 0.4	20
26	Mycophenolate	mRNA	< 0.4	25
27	Mycophenolate, Tacrolimus, Prednisone	mRNA	199	35
28	Mycophenolate, Tacrolimus, Prednisone	Vector	< 0.4	0
29	Fingolimod	mRNA	93	8.75
30	Mycophenolate, Tacrolimus, Prednisone	Vector	0.74	12.5
31	Mycophenolate, Tacrolimus	Vector	< 0.4	13
32	Mycophenolate, Tacrolimus	mRNA	2.75	2.5
33	Mycophenolate, Ciclosporin	mRNA	26.6	7.5

34	Mycophenolate, Ciclosporin	mRNA	< 0.4	114.5
35	Mycophenolate, Tacrolimus, Prednisone	mRNA	46.4	6.25
36	Mycophenolate, Tacrolimus, Prednisone	mRNA	325	5
37	Mycophenolate	Vector	< 0.4	15
38	Mycophenolate, Tacrolimus, Prednisone	mRNA	< 0.4	15
39	Mycophenolate, Sirolimus	Vector	< 0.4	46
40	Vinorelbine	mRNA	2.38	651
41	Mycophenolate, Tacrolimus	mRNA	< 0.4	107.5
42	Tacrolimus	Vector	< 0.4	36
43	Tacrolimus	Vector	< 0.4	22
44	Mycophenolate, Tacrolimus	Vector	< 0.4	27.5
45	Tacrolimus	mRNA	318	25
46	Cabozantinib	Vector	2.65	1.25

Immunosuppressive treatment, type of vaccine used and humoral as well as cellular immune response (sum of S1 and S2 peptide pools, SFC per 10^6 PBMCs) for each individual patient. IMiD: immunomodulatory imide drug, JAKi: Janus kinase inhibitor

Supplementary Methods

Interventions

Four study visits were performed. In the context of the screening visit (visit one), primarily immunosuppressive medication and possible previous SARS-CoV-2 infections were recorded. Furthermore, lack of antibody against spike and nucleocapsid protein was verified at screening, and peripheral blood leukocyte typing was performed. The additional vaccine dose was applied during the baseline visit (visit two, within 28 days after screening). Efficacy and safety were evaluated at visits three and four (one and four weeks after vaccination, respectively). The serum samples acquired from visit one, three and four were stored below -70°C at the Biobank of the Medical University of Vienna, a certified (International Organization for Standardization (ISO) 9001:2015) and centralized facility for the preparation and storage of biomaterial[1]. Additionally, at the screening visit and at visit two, peripheral blood mononuclear cells (PBMCs) were isolated by density gradient centrifugation and stored in the vapor phase of liquid nitrogen until further use.

Quantification of peripheral leukocytes.

Flow cytometry (FACSCanto II, Becton Dickinson, San Jose, California, USA) was utilized to determine the phenotype of peripheral leukocytes. Hereby staining of whole blood followed by lyse-wash procedure (Becton Dickinson) was performed. The following monoclonal antibodies were used to classify lymphocytes subpopulations (all provided by Becton Dickinson): fluorescein isothiocyanate (FITC)-labelled anti-CD3, phycoerythrin (PE)-labelled anti-$CD16^{+}56^{+}$, peridinin-chlorophyll-protein (PerCP)-cy5.5-labelled anti-CD4, PE-Cy7-labelled anti-CD19, allophycocyanin (APC)-Cy7-labelled anti-CD8, V450-labelled anti-human leukocyte antigen (HLA)-DR, V500-labelled anti-CD45 and APC-labelled anti-CD14. Results are presented either as absolute numbers or as percentage of a subpopulation among total lymphocytes.

Anti-SARS-CoV-2 antibody testing.

Quantification of antibodies to the receptor-binding domain (RBD) of the viral spike (S) protein was performed using the Elecsys Anti-SARS-CoV-2 S immunoassay[2,3]. The detection range is between 0.4 and 2500.0 BAU/mL (binding antibody units per milliliter). Antibody concentrations over 0.8 BAU/mL were considered positive. Analysis was performed on a Cobas e801 device (Roche Diagnostics, Rotkreuz, Switzerland) at the Department of Laboratory Medicine, Medical University of Vienna (certified acc. to ISO 9001:2015 and accredited acc. to ISO 15189:2012).

Determination of SARS-CoV-2 specific T-cell responses

For T-cell stimulation (see below), PepMix SARS-CoV-2 peptide pools were acquired from JPT (Berlin, Germany). The pools cover the entire sequences of the SARS-CoV-2 S protein and comprise 15-mer peptides overlapping by 11 amino acids (aa). The S peptides are split into two subpools S1 (aa 1–643) and S2 (aa 633–1273). Peptides were dissolved in dimethyl sulfoxide and diluted in AIM-V medium for use in enzyme-linked immunosorbent spot (ELISpot) assays.

For *ex vivo* T-cell IFN-γ ELISpot assay, PBMCs from patients before and after the third vaccination were thawed and processed on the same day. A total of $1–2\times10^5$ cells per well were incubated with SARS-CoV-2 peptides (2 µg/mL; duplicates), AIM-V medium (negative control; 3–4 wells) or phytohemagglutinin (PHA) (L4144, Sigma; 0,5 µg/mL; positive control) in 96-well plates coated with 1.5 µg anti-IFN-γ (1-D1K, Mabtech, 1:67) for 24 hours. After washing, spots were developed with 0.1 µg biotin-conjugated anti-IFN-γ (7-B6-1, Mabtech, 1:1000), streptavidin-coupled alkaline phosphatase (Mabtech, 1:1000) and 5-bromo-4-chloro-3-indolyl phosphate/nitro blue tetrazolium (Sigma). Spots were counted using a Bio-Sys Bioreader 5000 Pro-S/BR177 and Bioreader software generation 10. Data were calculated as spot-forming cells (SFCs) per 10^6 PBMCs after subtracting the spots from the negative control (mean spot numbers from three to four unstimulated wells). Samples where a PHA response < 600 SFC/10^6 PBMCs has been observed were excluded.

Determination of SARS-CoV-2 Spike-specific memory B-cells

For detection of SARS-CoV-2 Spike (S) protein-specific memory B-cells, biotinylated S protein antigen was individually mixed with streptavidin-APC and streptavidin-BV421 probes in similar fashion as in Dan et al[4]. Streptavidin-APC-Cy7 without biotinylated protein was used as a decoy probe to gate out B cells that non-specifically bind streptavidin. The S protein antigen probes and decoy probe were mixed in Brilliant Buffer (BD Bioscience, Cat# 566349) containing 5µM free D-biotin (to minimize potential cross-reactivity between probes) and for staining $3x10^6$ cryopreserved PBMC prepared in 96-well U-bottom plates were incubated with 50µL antigen probe cocktail, containing 100ng S protein per probe, at 4°C for one hour. Thereafter, surface staining was performed with directly-labeled monoclonal Abs towards human CD19 (FITC, clone HIB19), human CD27 (PE, clone L128), human CD38 (PerCP-Cy5.5, clone HIT2) and human immunoglobulin D (IgD) (PE-Cy7, clone IA6-2), all BD Bioscience, in Brilliant Buffer at 4°C for 30 min. Dead cells were excluded by using Fixable viability dye eFluor-506 (eBioscience, now Thermo Fisher Scientific). Data were acquired on a FACS Canto II flow cytometer by gating on cells with forward/side light scatter properties of lymphocytes and analyzed with FACS Diva 8.0 software. S-specific memory B-cells were quantified as percentages of total memory B-cells, including the $CD19^+$ un-switched ($IgD^+/CD27^+$), switched ($IgD^-/CD27^+$) and double-negative ($IgD-/CD27^-$) memory subset.

HLA-specific antibody testing

Sera were treated with EDTA (5mM final concentration) before the test to prevent the prozone phenomenon. The detection and specification of HLA-class I and class II-specific antibodies were performed using Single Antigen Beads (LS1A04-SA1 and LS2A01-SA2 OneLambda, West Hills, CA, USA), respectively, according to the manufacturer’s recommendations. Samples were measured on a Luminex FlexMap 3D (Luminex, Austin, TX, USA) and analyzed using the HLA Fusion software (OneLambda, V4.2).

Supplementary References

1. Haslacher, H. *et al.* Usage Data and Scientific Impact of the Prospectively Established Fluid Bioresources at the Hospital-Based MedUni Wien Biobank. *Biopreservation Biobanking* **16**, 477–482 (2018).
2. Higgins, V., Fabros, A. & Kulasingam, V. Quantitative Measurement of Anti-SARS-CoV-2 Antibodies: Analytical and Clinical Evaluation. *J. Clin. Microbiol.* **59**, e03149-20.
3. Perkmann, T. *et al.* Anti-Spike Protein Assays to Determine SARS-CoV-2 Antibody Levels: a Head-to-Head Comparison of Five Quantitative Assays. *Microbiol. Spectr.* e0024721 (2021) doi:10.1128/Spectrum.00247-21.
4. Dan, J. M. et al. Immunological memory to SARS-CoV-2 assessed for up to 8 months after infection. Science **371**, eabf4063 (2021).

Clinical Trial Protocol

Clinical Study Protocol

A Phase II Study to Evaluate Safety and Efficacy to a Third Vaccination in Immunocompromised Patients with Inadequate Humoral Response after Primary mRNA SARS-CoV-2 (Covid-19) Vaccination

Daniela Sieghart[1], Selma Tobudic[2], Martina Durechova[1], Helga Lechner-Radner[1], Peter Mandl[1], Daniel Mrak[1], Michael Bonelli[1], Daniel Aletaha[1]

1 Department of Internal Medicine III, Division of Rheumatology, Medical University of Vienna, Austria
2 Department of Medicine I, Division of Infectious Diseases and Tropical Medicine, Medical University of Vienna, Austria

Confidentiality Statement
The information contained in this document, especially unpublished data, is the property of the sponsor of this study. It is therefore provided to you in confidence as an Investigator, potential Investigator, or consultant, for review by you, your staff, and an Independent Ethics Committee or Institutional Review Board. It is understood that this information will not be disclosed to others without written authorization from the Sponsor except to the extent necessary to obtain informed consent from those persons to whom the study drug may be administered.

1. SPONSOR, INVESTIGATOR, MONITOR AND SIGNATURES

Sponsor/or representative (OEL) (AMG §§ 2a, 31, 32)

Univ.-Prof. Dr.med.univ. Alexandra Kautzky-Willer,

Department of Internal medicine III, Medical University of Vienna, Austria

Signature (OEL)	Date

Coordinating Investigator (AMG §§ 2a, 35, 36)

Univ.-Prof. Dr.med.univ. Daniel Aletaha,

Department of Internal Medicine III, Division of Rheumatology, Medical University of Vienna, Austria

Signature	Date

2. Study summary

Background. Immunosuppressive therapies are frequently used for treating patients with chronic inflammatory diseases such as rheumatoid arthritis, SLE or other connective tissue diseases, vasculitides, spondyloarthropathies, psoriasis, and inflammatory bowel diseases or patients with inflammatory neurological diseases, such as multiple sclerosis, malignant diseases or solid organ transplantation, etc. Yet the efficacy of mRNA vaccination against severe acute respiratory syndrome coronavirus 2 (SARS-CoV-2) in patients treated with glucocorticoids, disease-modifying antirheumatic drugs or other immunosuppressive therapies may be impaired. In addition, patients with immunodeficiencies are also prone to show reduced response to vaccination. It is unclear how to proceed with immunocompromised individuals who did not show an adequate response to standard vaccination protocols (no or low titres of antibodies).

Objective. To investigate if a third shot of the SARS-CoV-2 vaccine can lead to humoral immune response in immunocompromised individuals who did not respond to the standard vaccination schedule.

Methods. We will perform a prospective study of two parts. In part A a total of 150 patients under current immunosuppressive treatment and without response to standard vaccination with a SARS-CoV-2 vaccine (Biontech/Pfizer or Moderna) will be enrolled in this randomised single blinded controlled clinical trial receiving a second boost vaccination with either a mRNA vaccine (dependent on the standard vaccine) or a vector-based vaccine (Astra Zeneca). In part B, 75 immunocompromised patients (immunosuppressed or immunodeficient) as well as 75 healthy controls with low titres after standard vaccination will be enrolled to receive a third mRNA vaccination. Four study visits per patient will be planned. During the screening visit antibodies to the receptor-binding domain will be determined. After vaccination (baseline visit) additional study visits are scheduled at weeks 1 and 4 post vaccination for assessment of humoral and cellular immunity, as well as clinical signs of adverse effects of disease activity reactivation. Patients will be offered participation in an extended follow-at week 12 after vaccination.

Expected Results. Part A: we expect at least 40% of patients without prior humoral response receiving a third SARS-CoV-2 vaccination to seroconvert after 4 weeks. The change in vaccine modality is expected to have a benefit over a third vaccination with an mRNA vaccine. Part B: we expect that 70% immunocompromised patients as well as healthy controls with low titre antibody show an adequate response (>1.500 U/ml SARS-CoV-2 antibody titre) to a third vaccination with a mRNA SARS-CoV-2 vaccine. The rate of responders of immunocompromised patients is expected to be non inferior to those of healthy controls.

3. BACKGROUND

The current pandemic caused by severe acute respiratory syndrome coronavirus-2 (SARS-CoV-2) has led to exponentially rising morbidity and mortality worldwide. Apart from aggressive quarantine and hygiene control measures, the most effective way to inhibit SARS-CoV-2 spread is a population-wide vaccination campaign.
Two mRNA vaccines, BNT162b2, developed by BioNTech/Fosun Pharma/Pfizer, and mRNA-1273 developed by Moderna/NIAID, are currently in phase III trials.[1,2]

Preliminary results from Phase I–II clinical trials on BNT162b2, published in October 2020, indicated the potential for its efficacy and safety.[3,4] Findings from studies conducted in the United States and Germany among healthy men and women showed that two 30-μg doses of BNT162b2 elicited high SARS-CoV-2 neutralizing antibody titers and robust antigen-specific CD8+ and Th1-type CD4+ T-cell response.[5]
In July 2020, preliminary results of the Phase I dose-escalation clinical trial of mRNA-1273 were published, showing dose-dependent induction of neutralizing antibodies against SARS-CoV-2 as early as 15 days post-injection.[1] Preliminary data from Phase III clinical trial, published in November 2020, indicating 94% efficacy in preventing COVID-19 infection.

There are no data about the efficacy and safety of mRNA vaccines against SARS-CoV-2 in immunocompromised patients (i.e. with a chronic immune-mediated disease such as various rheumatological, hematological, neurological, and other conditions like patients who are post-transplantation and receive immunosuppressive therapy or patients with

immunodeficiencies). Moreover, it is not clear whether mRNA vaccination against SARS-CoV-2 in immunocompromised patients has a protective effect at all.

Therefore, clinical studies on the characterization of humoral immunity after vaccination against SARS-CoV-2 in immunocompromised patients are urgently needed.

In addition to mRNA vaccines the ChAdOx1 nCoV-19 vector-based vaccine was developed by AstraZeneca and received admission by the by the European Commission on January the 29th 2020 after recommendation of the European Medicines Agency (EMA). The vaccine uses a chimpanzee adenoviral vector. It delivers the gene that encodes the SARS-CoV-2 spike protein. The vaccine was shown to be effective during two phase III trials in the United Kingdom and Brazil.[6] The overall efficacy in preventing symptomatic infection more than 14 days after the second dose was 70.4% (95%CI: 54.8%-80.6%), with efficacy of 62.1% (95%CI: 41.0%-75.7%) in those who received standard doses, and 90.0% (95%CI 67.4% to 97.0%) in those who received a half-dose followed by a standard dose.

Immunocompromised patients with COVID-19 may be at higher risk of hospitalization and ICU admission than matched comparators.[7] A vaccination with an mRNA vaccine should be performed as primary immunization with two vaccine-doses 21 or 28 days apart.
As recently shown patients under immunosuppressive therapy have a reduced seroconversion rate compared to healthy subjects [8]

It is unclear if humoral or cellular immunity to SARS-CoV-2 can be induced by a second boost vaccination with either an mRNA vaccine or if patients would benefit from a change in the mode of action of the applied vaccine using a vector-based vaccine.

4. STUDY RATIONALE

Immunocompromised patients might have an increased risk of non-response or reduced efficacy to SARS-CoV-2 vaccination. It is unclear whether patients who did not develop humoral immunity after a standard protocol application with a SARS-CoV-2 vaccine would benefit from a second boost vaccination of the same mode of action or from a single additional shot of cross vaccination against SARS-CoV-2. Furthermore, it is unclear if

immunocompromised patients who developed only low titre antibodies against SARS-CoV-2 would benefit form a third mRNA vaccination and if response to vaccination is non inferior compared to those of a healthy control group.

5. STUDY OBJECTIVES

The study aims to investigate the humoral and cellular immune responses after a second boost vaccination against SARS-CoV-2 in adult immunocompromised patients who did not show or had only little response to the standard vaccination. Therefore the study will be conducted in two parts, assessing two different primary objectives: For Part A, in patients under immunosuppressive therapy without detectable SARS-CoV-2 antibodies a heterologous versus homologous vaccination scheme will be tested. For Part B, patients under immunosuppressive therapy or with immunodeficiencies as well as healthy controls with low antibody titres will receive an additional booster vaccination and immune response will be compared across the two groups.

5.1. Primary Objective (Hypothesis)

PART A) To assess the immunogenicity to a third vaccination mRNA-SARS-CoV-2 vaccine (Biontech/Pfizer or Moderna) compared to a vector SARS-CoV-2 (AstraZeneca) vaccination as a second boost in patients with immunosuppressive therapy without humoral response to the standard vaccination by measuring quantitative antibody levels by spike-protein-based assay. Patients who developed detectable antibodies against SARS-CoV-2 will be defined as responders.

Null and alternative hypotheses:

H0: There is no statistical difference in the seroconversion rate between patients receiving a third mRNA vaccination and the patients receiving a second boost with a vector vaccine.

H1: There is statistical difference in the seroconversion rate between patients receiving a third mRNA vaccination and the patients receiving a second boost with a vector vaccine.

PART B) To compare the immunogenicity to a third vaccination with a mRNA-SARS-CoV-2 vaccine as a second boost in immunocompromised patients and healthy controls who developed insufficient titres of antibodies (< 1500 U/ml) after the standard vaccination by measuring quantitative antibody levels by spike-protein-based assay. The level of antibody increase will be compared between the two groups.

Null and alternative hypotheses:

H0: There is a statistical difference in the percentage of participants achieving an adequate response (>1.500 U/ml SARS-CoV-2 antibody titre) after a second boost between patients and healthy controls after receiving a second boost with a mRNA vaccine.

H1: There is no statistical difference in the percentage of participants achieving an adequate response (>1.500 U/ml SARS-CoV-2 antibody titre) after receiving a second boost with a mRNA vaccine and the rate of responders of immunocompromised patients is non inferior to those of healthy controls.

Secondary Objectives

- Cellular immunogenicity of the third mRNA SARS-CoV-2 vaccination will be compared to patients receiving a vector vaccination as second boost in immunocompromised patients without prior humoral response. Further, T cell responses will be compared in immunocompromised patients as well as healthy controls before and after a second mRNA boost vaccination. T cell proliferation will be assessed, and T-cell cytokine expression will be measured using flow-cytometry following *in vitro* stimulation of peripheral blood mononuclear cells (PBMCs) with SARS-CoV-2 specific antigens.

- Comparison of total antibody titre after the second boost as well as absolute and relative change in antibody titre before and after the second boost in immunosuppressed patients versus the healthy controls depending on

  - titre level after second immunization
  - presence of B-cells

- type of immunosuppressive agent
- concomitant steroid therapy
- type of underlying rheumatic disease
- patient characteristics (age, gender, time between second and third vaccination)

- To assess safety of a second boost vaccination.
- To qualitatively evaluate the influence of a second boost vaccination on underlying disease treated with immunosuppressive therapy. Patients will be asked to complete a patient´s diary for up to seven days after vaccination including information on the change/worsening of their underlying disease.

6. STUDY DESIGN

A prospective single blind randomized controlled study will be performed. A total of 300 immunocompromised patients or healthy subjects will be enrolled in this clinical trial. Four study visits per participant will be planned.

Immunosuppressive therapies can be applied for different types of diseases like autoimmune diseases including RA, SLE or MS, malignancies as well as to reduce the risk of graft rejection after organ transplantation. Immunosuppressive drugs target different parts of the immune system. They can be divided in 5 groups: glucocorticoids, cytostatics, antibodies, drugs acting on immunophilins and other treatments like interferons and TNF binding proteins. The type of immunosuppression is not limited, however, B-cell depleting therapies like Rituximab, Ocrelicumab, Ofatumumab, Epratuzumab or Obinutuzumab) will be excluded. Patients with primary or secondary immunodeficiency are also considered immunocompromised and therefore included in the study.

For Part A stratification will be performed by the number of B cells determined at screening visit.

Medication and medical history will be collected in a concomitant medication log and a medical history log.

After inclusion of the participants (after receiving their written informed consent), serum samples for determination of antibody levels will be obtained at least 4 weeks after the

second mRNA vaccination (screening visit). Information on immunization dates of patients as well as previous infections with Covid-19 will be collected during the screening visit. All patients who fulfill the inclusion criteria and do not fulfill any exclusion criteria will be invited for a second boost vaccination within 4 weeks after the screening visit. Randomization is only applicable for patients without detectable humoral response to SARS-CoV-2 after standard vaccination (Part A). Additional study visits are scheduled at baseline (vaccination), and at weeks 1 and 4 for assessment of humoral and cellular immunity, as well as clinical signs of adverse effects of disease activity reactivation. Patients will be offered participation in an extended follow-up for additional 8 weeks (12 after baseline).

Part A:

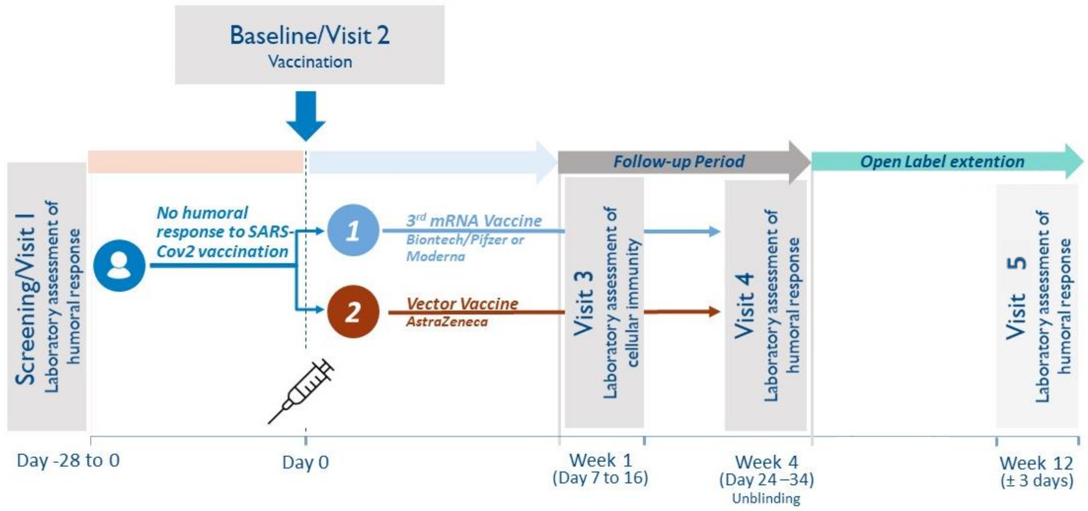

Part B:

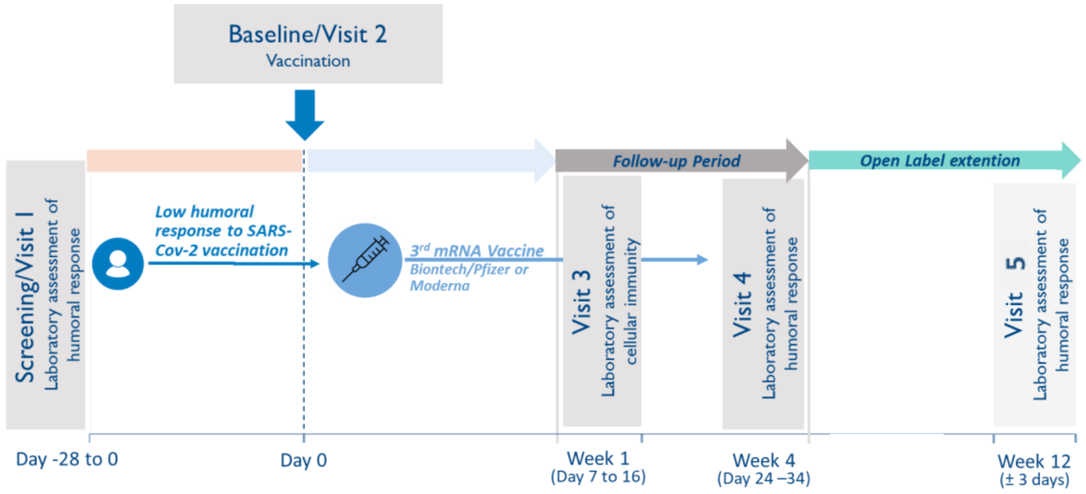

6.1. Study population

Adult immunocompromised patients (≥ 18 years) who did develop no or inadequate (< 1500 U/ml) levels of antibodies against SARS-CoV-2 after their standard vaccination will be recruited at the Division of Rheumatology, Medical University of Vienna. For Part B, a control group of healthy participants, also with low titers (<1500) will be included.

Study entry is defined as the date of signature of the study participant (subject) on the informed consent form. All subjects enrolled will be assigned a subject code, consisting of a consecutive subject number (3 digits).

6.2. Inclusion criteria

a) Male and female patients will be eligible for participation in this study if they:

1. Are ≥18 years on the day of screening
2. Being immunocompromised (either primary or secondary immunodeficiency or been treated with immunosuppressive therapy within the last 12 months

   Immunosuppressive therapies include glucocorticoids, cytostatics, antibodies, drugs acting on immunophilins and other treatments like interferons and TNF binding proteins and exclude patients under B cell depleting therapy (like Rituximab, Ocrelicumab, Ofatumumab, Epratuzumab or Obinutuzumab)

3. Received two doses of SARS-CoV-2 (Biontech/Pfizer, Moderna) vaccine according to recommendations in the label and/or national guidelines.
4. Did develop < 1500 U/ml of SARS-CoV-2 antibodies 4 weeks after second mRNA vaccination (analyzed during the study “Characterization of immune responsiveness after mRNA SARS-CoV-2 Vaccination in patients with immunodeficiency or immunosuppressive therapy”, EK-Nr. 1073/2021, EudraCT Nr. 2021-000291-11, or external routine evaluation of humoral response)
5. A maximum of 12 months after second vaccination
6. Have an understanding of the study, agree to its provisions, and give written informed consent before study entry
7. If female and capable of bearing children – have a negative urine pregnancy test result at study entry and agree to employ adequate birth control measures for the duration of the study

b) Male and female healthy controls will be eligible for participation in this study if they:

1. Are ≥18 years on the day of screening
2. Received two doses of SARS-CoV-2 (Biontech/Pfizer, Moderna) vaccine according to recommendations in the label and/or national guidelines.
3. Did develop < 1500 U/ml of SARS-CoV-2 antibodies 4 weeks after second mRNA vaccination
4. A maximum of 12 months after second vaccination
5. Have an understanding of the study, agree to its provisions, and give written informed consent before study entry
6. If female and capable of bearing children – have a negative urine pregnancy test result at study entry and agree to employ adequate birth control measures for the duration of the study

6.3. Exclusion criteria

Subjects will be excluded from participation in this study if they:

1. Have shown humoral response (> 1500 U/ml) to the SARS-CoV-2 vaccination
2. Had grade 3 adverse effects from the mRNA vaccination reported
3. Pregnancy and breast feeding
4. Signs of SARS-CoV-2 infection (including previous positive PCR testing)
5. Any other contraindication to any of the vaccine compounds
6. For healthy controls: diagnosis of chronic inflammatory condition including primary or secondary immunodeficiency or ever receiving immunosuppressing therapy as stated above

6.4. Study duration

For the individual study participant, the active study phase will be 4 weeks, with possibility to enroll in an extension for additional 12 weeks.

6.5. Randomization Procedure (only Part A)

The web-based computerized randomization algorithm by randomlists.com will be used for randomization. Patients will be randomized in a 1:1 ratio between the third dose mRNA

SARS-CoV-2 boost (Biontech/Pfizer or Moderna, respective of their initial vaccination compound and Biontech/Pfizer for patients immunized with Astra Zeneca) and a single dose boost vector SARS-CoV-2 vaccine (AstraZeneca).

Block randomization according to number of peripheral B-cells (determined at screening visit) will be performed.

For Part B no randomization process will take place.

6.6.1. Blinding

Patients without prior humoral response to SARS-CoV-2 (only Part A) will be blinded. Blinding of initial treatment allocation will be maintained throughout the 4 week study period. Blinding is ensured during the routine vaccination protocol at the General Hospital Vienna, where patients will see the pre-arranged dose aliquots in syringes without reference to the type of vaccine. To facilitate the process of vaccination of full vials, patients randomized to the same vaccine will be scheduled on the same day. Blinding will be mainly performed to prevent selective drop-outs due to knowledge of treatment allocation.

6.6.2. Unblinding Process

The blinding can be lifted at the request of an investigator, when knowledge of the treatment is essential for appropriate patient management (e.g. after trial termination). In case of an emergency, the principal investigator has the sole responsibility for determining if unblinding of a patient´s treatment assignment is warranted.

If any serious adverse event arises during the study and unblinding appears necessary, the sub investigator will notify the principal Investigator within two days in maximum. In the following the matter will be discussed and may lead to unblinding.

6.7.1 Withdrawal and replacement of subjects

Criteria for withdrawal:

Subjects may prematurely discontinue from the study at any time. The study's premature discontinuation is to be understood when the subject did not undergo complete the last visit (study visit 4) or all pivotal assessments during the study.

Subjects must be withdrawn under the following circumstances:

- at their own request
- if the investigator feels it would not be in the best interest of the subject to continue
- if the subject violates conditions laid out in the consent form/information sheet or disregards instructions by the study personal

In all cases, the reason why subjects are withdrawn must be recorded in detail in the CRF and the subject's medical records. Should the study be discontinued prematurely, all study materials (complete, partially completed, and empty CRFs) will be retained.

Follow-up of patients withdrawn from the study:

In case of premature discontinuation after the start of vaccination, no further investigations concerning the study will be performed. Furthermore, participants may request that no more data will be recorded from the time point of withdrawal and that all biological samples collected in the course of the study will be destroyed.

6.7.2 Premature termination of the study

The sponsor has the right to close this study at any time. The IEC and the competent regulatory authority must be informed within 15 days of early termination.

The trial or single-dose steps will be terminated prematurely in the following cases:

- If the number of drop-outs is so high that proper completion of the trial cannot realistically be expected.
- Recruitment is not reaching the critical number

6.8. Adverse events and reporting

a. Definition of adverse events

An adverse event is any untoward medical occurrence in a patient or clinical investigation subject administered a pharmaceutical product and which does not necessarily have a causal relationship with this treatment. An adverse event (AE) can therefore be any unfavorable and unintended sign (including an abnormal laboratory finding), symptom, or disease temporally associated with the use of a medicinal (investigational) product, whether or not related to the medicinal (investigational) product (see the ICH Guideline for Clinical Safety Data Management: Definitions and Standards for Expedited Reporting)

Adverse events include:

- Exacerbation of a pre-existing disease.

- Increase in frequency or intensity of a pre-existing episodic disease or medical condition.
- Disease or medical condition detected or diagnosed after study drug administration even though it may have been present prior to the start of the study.
- Continuous persistent disease or symptoms present at baseline that worsen following the start of the study.
- Lack of efficacy in the acute treatment of a life-threatening disease.
- Events considered by the Investigator to be related to study-mandated procedures.
- Abnormal assessments, e.g., ECG and physical examination findings, must be reported as AEs if they represent a clinically significant finding that was not present at baseline or worsened during the course of the study.
- Laboratory test abnormalities must be reported as AEs if they represent a clinically significant finding, symptomatic or not, which was not present at baseline or worsened during the course of the study or led to dose reduction, interruption or permanent discontinuation of study drug.

Adverse events do not include:

- Pre-planned interventions or occurrence of endpoints specified in the study protocol are not considered AE´s, if not defined otherwise (eg.as a result of overdose)
- Medical or surgical procedure, e.g., surgery, endoscopy, tooth extraction, transfusion. However, the event leading to the procedure is an AE. If this event is serious, the procedure must be described in the SAE narrative.
- Pre-existing disease or medical condition that does not worsen.
- Situations in which an adverse change did not occur, e.g., hospitalisations for cosmetic elective surgery or for social and/or convenience reasons.

Overdose of either study drug or concomitant medication without any signs or symptoms. However, overdose must be mentioned in the Study Drug Log.

b. Definition of serious adverse events (SAEs)

A Serious Adverse Event (SAE) is defined by the International Conference on Harmonization (ICH) guidelines as any AE fulfilling at least one of the following criteria:

- Fatal (including fetal death).

- Life-threatening – defined as an event in which the subject was, in the judgment of the investigator, at risk of death at the time of the event; it does not refer to an event that hypothetically might have caused death had it been more severe.
- Requiring subject's hospitalization or prolongation of existing hospitalization – inpatient hospitalization refers to any inpatient admission, regardless of length of stay.
- Resulting in persistent or significant disability or incapacity (i.e., a substantial disruption of a person’s ability to conduct normal life functions).
- Congenital anomaly or birth defect.
- Is medically significant or requires intervention to prevent at least one of the outcomes listed above.

In case of any SAE, the investigator has to use all means to ensure patient´s safety. Every SAE has to be reported as outlined in section 16.8.

c. Definition of suspected or unexpected serious adverse reactions (SUSARs)

SUSARs are all suspected adverse reactions related to the study drug that are both unexpected (not previously described in the SmPC or Investigator’s brochure) and serious.

d. Pregnancy

Maternal pregnancies must be reported to the principal investigator/sponsor. To ensure subject safety, each pregnancy must be reported to the PI/sponsor within 2 weeks of learning of its occurrence.

Pregnancies, should be followed up and reported to the PI/sponsor until the outcome of the pregnancy (including premature termination) and status of mother and child is known.

e. Severity of adverse events

The severity of clinical AEs is graded on a three-point scale: mild, moderate, severe, and reported on specific AE pages of the CRF.

If the severity of an AE worsens during study drug administration, only the worst intensity should be reported on the AE page. If the AE lessens in intensity, no change in the severity is required:

- *Mild* - Event may be noticeable to subject; does not influence daily activities; the AE resolves spontaneously or may require minimal therapeutic intervention;

- *Moderate* - Event may make subject uncomfortable; performance of daily activities may be influenced; intervention may be needed; the AE produces no sequelae.
- *Severe* - Event may cause noticeable discomfort; usually interferes with daily activities; subject may not be able to continue in the study; the AE produces sequelae, which require prolonged therapeutic intervention.

A mild, moderate or severe AE may or may not be serious.

f. Relationship of adverse events to study drug

For all AEs, the investigator will assess the causal relationship between the study drug and the AE using his/her clinical expertise and judgment according to the following algorithm that best fits the circumstances of the AE:

Not related

- May or may not follow a temporal sequence from administration of the study product
- Is biologically implausible and does not follow known response pattern to the suspect study drug (if response pattern is previously known).
- Can be explained by the known characteristics of the subject's clinical state or other modes of therapy administered to the subject.

Unlikely

- There is a reasonable temporal relation between the AE and the intake of the study medication, but there is a plausible other explanation for the occurrence of the AE.

Possibly

- The AE has a reasonable temporal relationship with drug administration.
- The AE may equally be explained by the study subject's clinically state, environmental or toxic factors, or concomitant therapy administered to the study subject.
- The relationship between study drug and AE may also be pharmacologically or clinically plausible.

Probably

- There is a reasonable temporal relation between the AE and the intake of the study medication, and plausible reasons point to a causal relation with the study medication.

Related

- Reasonable temporal relation between the AE and the intake of the study medication and
- There is no other explanation for the AE and
- Subsidence or disappearance of the AE on withdrawal of the study medication and
- Recurrence of the symptoms on restart at previous dose (only applies for re-institution of mediation).

Not assessable

The causal relationship between the study drug and the AE cannot be judged.

g. Adverse events reporting procedures

A special section is designated to adverse events in the case report form. For each subject, adverse events occurring after signing the informed consent must be recorded on the applicable adverse events page(s) in the case report form. Recording should be done in a concise manner using standard, acceptable medical terms. The adverse event recorded should not be a procedure or a clinical measurement (i.e., a laboratory value or vital sign) but should reflect the reason for the procedure or the diagnosis based on the abnormal measurement. If, in the investigator's judgment, a clinically significant worsening from baseline is observed in laboratory or other parameters, physical exam finding, or vital sign, a corresponding clinical adverse event should be recorded on the adverse event page(s) of the CRF. If a specific medical diagnosis has been made that diagnosis should be recorded on the adverse event page(s) of the CRF.

SAEs and any newly identified pregnancy (maternal or paternal exposure), malignancy, overdose, opportunistic infection or case of active TB occurring after first administration of study agent in subjects participating in this clinical trial requires submission of a Safety Report Form to the Sponsor (only if other sites are reporting SAE) within 24 hours of notification or observation.

The following details must thereby be entered:

- Type of adverse event
- Start (date and time)
- End (date and time)
- Severity (mild, moderate, severe)

- Serious (no / yes)
- Unexpected (no / yes)
- Outcome (resolved, ongoing, ongoing – improved, ongoing – worsening)
- Relation to study drug (unrelated, possibly related, definitely related)

Adverse events are to be documented in the case report form in accordance with the above mentioned criteria.

h. Reporting procedures for SAEs

In the event of serious, the investigator has to use all supportive measures for best patient treatment. A serious adverse event must be reported if it occurs during a subject's participation in the study (whether receiving study agent or not) or within six (6) months of receiving the last dose of study agent.

A Safety Report Form must be completed and faxed to the Sponsor (+43 (0)1 40400 43060) 24 hours of observation or notification of the event. The sponsor will be responsible for potential SUSAR assessment (see below) and reporting SAEs including potential SUSAR to the manufacturing company. A written report is also to be prepared and made available to the clinical investigator within five days.

The following details should at least be available:

- Patient initials and number
- Patient: date of birth, sex, ethical origin
- The suspected investigational medical product (IMP)
- The adverse event assessed as serious
- Short description of the event and outcome

Any serious adverse event that is ongoing when a subject completes his/her participation in the trial must be followed until any of the following occurs:

- The event resolves or stabilizes
- The event returns to baseline condition or value (if a baseline value is available)
- The event can be attributed to agent(s) other than the study agent, or to factors unrelated to study conduct

i. Reporting procedures for SUSARs

All SAEs will be evaluated regarding a possible classification as SUSAR by the sponsor, who will then perform all necessary reports to the manufacturing/distributing pharmaceutical company and forward the SUSAR to the CRO. The CRO will be responsible for reporting SUSARs to the regulatory authorities. In addition, due to their possible safety concern for the study participants, all SUSARs need to be reported to the Institutional Review Board / Independent Ethics Committee (IRB / IEC).

These reports are time critical and should be done within a maximum of 7 days (fatal or life threatening outcome) or 15 days (non fatal, not life threatening). The representative of the Sponsor Investigator shall inform all investigators concerned of relevant information about Serious Unexpected Suspected Adverse Reactions that could adversely affect the safety of subjects.

Such reports shall be made by the study management and the following details should be at least available:

- Patient inclusion number
- Patient: year of birth, sex, ethical origin
- Name of investigator and investigating site
- Period of administration
- The suspected investigational medical product (IMP)
- The adverse event assessed as serious and unexpected, and for which there is a reasonable suspected causal relationship to the IMP
- Concomitant disease and medication
- Short description of the event:
    - Description
    - Onset and if applicable, end
    - Therapeutic intervention
    - Causal relationship
    - Hospitalization of prolongation of hospitalization

METHODOLOGY

7.1 Study design and blood samples

In Part A, a randomized controlled single-blind phase II trial will be performed. For Part B a prospective open label trial will be performed. A total of 300 participants (immunocompromised patients and healthy controls) who did develop no or low (< 1500 U/ml) humoral immune response after standard vaccination with a SARS-CoV-2 vaccine (Biontech/Pfizer, Moderna). Four study visits per patient will be planned during the course of the trial: screening visit (-0-14 days), baseline visit (date of vaccination), week 1, week 4.

Patients participating in Part A with no detectable antibodies at screening visit will be blinded and randomized to receive either a third mRNA vaccination or a vector based vaccine and will be unblinded at week 4. Immunocompromised patients and healthy controls with inadequate titre (<1500 U/ml) participating in Part B will receive a third mRNA vaccination. In addition, participants will be invited for an additional study visit at week 12 after vaccination.

Blood samples for the determination of SARS-CoV-2 antibodies will be obtained at screening visit, at week 1, and week 4 and optional week 12.

7.2 Study procedures

7.2.1 General rules for trial procedures

- All study measures like blood sampling and measurements have to be documented with the date (dd:mm:yyyy).
- In case several study procedures are scheduled simultaneously, there is no specific sequence that should be followed.
- The dates of all procedures should be according to the protocol. The time margins mentioned in the study flow chart are admissible. If for any reason, a study procedure is not performed within scheduled margins, a protocol deviation should be noted, and the procedure should be performed as soon as possible or as adequate.
- If it is necessary for organizational reasons, it is admissible to perform procedures scheduled for one visit at two different time points. Allowed time margins should thereby not be exceeded.

7.2.2 Study visits

7.2.2.1 Screening visit (visit 1, -1-28 days, at least 4 weeks after the second immunization with mRNA SARS-CoV-2 vaccine)

The investigator will inform the subject about the procedures, risks, and benefits of the study. Participants will be informed about future visits, which will be synchronized with the appointment for the third vaccination.

Fully informed, written consent must be obtained from each subject before any assessment is performed. The subject must be allowed sufficient time to consider his/her participation in the study.

The following assessments will be performed:

- Inclusion and exclusion criteria
- Demographic data, including sex, age, weight, and height
- Medical history and concomitant medication
- Assessment of adverse reactions after the mRNA SARS-CoV-2 vaccination
- Assessment of recent COVID-19 disease
- Blood will be drawn:
    - 16 ml of blood will be drawn for PBMC isolation and determination of cellular immunity
    - 8 ml of blood will be drawn for SARS-CoV-2 antibody level detection (prior vaccination).
    - 8 ml of blood will be drawn for determination of leukocyte subpopulations
    - 8 ml of blood will be drawn for biobanking of serum
    - 20 ml of blood will be drawn for routine laboratory testing: blood count, chemistry, coagulation factors
    - 8 ml of blood will be drawn for QuantiFERON assay

- Pregnancy test in women with childbearing potential
    - Part A) Randomization to one of the three vaccines: mRNA SARS-CoV-2 (Biontech/Pfizer or Moderna) or vector SARS-CoV-2 vaccination (AstraZeneca)
- SARS-CoV-2 vaccine appointment

7.2.2.2. Baseline visit (visit 2, week 0, max. 28 days after visit 1)

The following activities will be performed:

- Check qualification of study participation (inclusion and exclusion criteria)
- The investigator will ask all subjects about any adverse experiences occurring since Visit 1 (screening). All adverse experiences will be documented in the CRF.
- Patients will receive a patients diary, fever thermometer and spacer
- Application of 3rd SARS-CoV-2 vaccination (depending on randomization Part A)

7.2.2.3. Visit 3 (1 week after the third SARS-CoV-2 vaccination)

The following activities will be performed:

- The investigator will ask all subjects about any adverse experiences occurring since Visit 2. All adverse experiences will be documented in the CRF.
- Patient´s diaries will be discussed
- Blood will be drawn:
    - 16 ml of blood will be drawn for PBMC isolation and determination of cellular immunity
    - 8 ml of blood will be drawn for SARS-Cov-2 antibody level detection
    - 8 ml of blood will be drawn for biobanking of serum
    - 20 ml of blood will be drawn for routine laboratory testing: blood account, chemistry, coagulation factors
    - 8 ml blood will be drawn for detection of anti-PDE4D antibody
    - 8 ml of blood will be drawn for QuantiFERON assay

7.2.2. 4. Visit 4 (4 weeks after the third SARS-CoV-2 vaccination)

The following activities will be performed:

- The investigator will ask all subjects about any adverse experiences occurring since Visit 3. All adverse experiences will be documented in the CRF.
- Patient´s diary will be returned to investigator
- Blood draw:
    - 8 ml of blood will be drawn for SARS-Cov-2 antibody level detection
    - 8 ml of blood will be drawn for biobanking of serum
    - 20 ml of blood will be drawn for routine laboratory testing: blood account, chemistry, coagulation factors
    - 8 ml blood will be drawn for detection of anti-PDE4D antibody

7.2.2.5. Visit 5 (week 12) (only for patients enrolling in open label extension)

The following activities will be performed:

- The investigator will ask all subjects about any adverse experiences occurring since Visit 3. All adverse experiences will be documented in the CRF.
- Blood draw:
    - 8 ml of blood will be drawn for SARS-CoV-2 antibody level detection
    - 8 ml of blood will be drawn for biobanking of serum
    - 16 ml of blood will be drawn for PBMC isolation

7.2.3 Determination of the humoral and cellular immunity

7.2.3.1 Determination of serum antibodies against vaccination antigens

Analysis will be performed at the Department of Laboratory Medicine, Medical University of Vienna. All procedures will be carried out according to standard operating procedures in an ISO 9001:2015 certified environment (ref: 10.1089/bio.2018.0032).

Previous SARS-CoV-2 infection will be detected using nucleocapsid-based chemiluminescence assays (e.g., Roche SARS-CoV-2 NC total antibody ECLIA, Abbott SARS-CoV-2 NC IgG CMIA). Vaccination response will be assessed by spike-protein-based assays (e.g., Technozym RBD ELISA, Siemens RBD immunoassay, Roche SARS-CoV-2 RBD ECLIA).

All analyses will be carried out at the Department of Laboratory Medicine, Medical University of Vienna.

Neutralization assays will be performed as described earlier.[9]

7.2.3.2 Determination of cellular immune response

To investigate cellular immunity following SARS-CoV-2 vaccination Peripheral blood mononuclear cells (PBMCs) will be isolated from heparinized venous blood by density gradient centrifugation at 400 *g* of heparinized blood over LSM 1077 Lymphocyte Separation Medium, cryopreserved and stored in liquid nitrogen for later use.

7.3 Study endpoints

7.3.1 Primary study endpoints

Part A) For patients without prior detectable humoral immune response to SARS-CoV-2 (<0.4 U/ml) the difference in SARS-CoV-2 antibody seroconversion rate by week 4 after vaccination boost at baseline between 3rd mRNA SARS-CoV-2 (Biontech/Pfizer or Moderna) and vector SARS-CoV-2 vaccine (AstraZeneca) will be assessed. Patients who develop detectable antibodies against SARS-CoV-2 will be defined as responders.

Part B) The efficacy of a 3rd mRNA boost vaccination in immunocompromised patients with inadequate humoral response (antibody titre <1500U/ml) to standard SARS-CoV-2 vaccination will be compared to low titre heathy controls by assessing the difference in antibody titre change before and after a second boost between the two groups.

7.3.2 Secondary study endpoints

The secondary endpoints of this study are:

- Overall SARS-CoV-2 antibody seroconversion rate by week 4 after vaccination boost in patients with no detectable antibodies at baseline
- Antibody concentrations including neutralizing antibodies against SARS-CoV-2 4 weeks after vaccination boost at baseline
- Difference in absolute and relative change of antibody titres before and after second mRNA vaccine boost between patients and healthy controls
- Effect of disease entity on SARS-CoV-2 antibody seroconversion rate and titre changes by week 4 after vaccination boost at baseline
- Effect of immunosuppressive medication and steroids on SARS-CoV-2 antibody seroconversion rate/titre change by week 4 after vaccination boost at baseline
- Effect of patient characteristics (age, gender, time between second and third vaccination) on seroconversion rate/titre change by week 4 after vaccination boost at baseline
- Evaluation of cellular immunity before and one week after the vaccination in all groups
- Safety of vaccination boost (all groups)
- Effect of vaccination on disease activity of underlying rheumatic disease

8. ETHICAL CONSIDERATIONS

a. Ethical Review

All relevant documents must be reviewed and approved by the Ethics Committee and additionally submitted to the relevant authorities in accordance with the guidance of submission and conduct of clinical trials.

The ethical committee of the Medical University Vienna will be asked to act as a 'Leitethikkommission'.

The clinical trial shall be performed in full compliance with the legal regulations according to the Drug Law (AMG - Arzneimittelgesetz) of the Republic of Austria.

An application must also be submitted to the Austrian Competent Authorities (Bundesamt für Sicherheit im Gesundheitswesen (BASG) represented by the Agency for Health and Food Safety (AGES PharmMed), and registered to the European Clinical Trial Database (EudraCT) using the required forms. The timelines for (silent) approval set by national law must be followed before starting the study.

b. Consent Procedures

After a detailed information about the study procedures and study medication, as well as the potentially related risks and benefits, the written informed consent will be obtained from each participant by the principal investigator or a designee. At each site, informed consent will be prepared according to the institutional requirements for informed consents. Consent will be collected by the investigator before patient inclusion in trial and before participating in any study procedure.

One copy of each consent form, signed by the participant and by the investigator, will be given to the patient and the original will be kept by the investigator.

c. Privacy of participants

All records will be kept confidential. The participant´s name will not be released at any time and data sets for each subject will be identified only by the patient enrollment number.

d. Amendments

Proposed amendments must be submitted to the appropriate Competent Authorities (CA) and Ethics Committee (EC) and approved before the change is implemented. These changes are usually presented in the form of an amendment. Amendments that are intended to eliminate an apparent immediate hazard to subjects may be implemented prior

to receiving CA/EC approval. However, in this case, approval must be obtained as soon as possible after implementation.

e. Insurance

Participants will be covered by a clinical trial insurance. The participants will be insured as defined during their participation in the clinical trial by legal requirements. The investigator of the clinical trial will receive a copy of the insurance conditions of the ´particpants insurance´. The sponsor is providing insurance in order to indemnify (legal and financial coverage) the investigator/center against claims arising from the study, except for claims that arise from malpractice and/or negligence. The compensation of the subject in the event of study-related injuries will comply with the applicable regulations. Details on the existing participants insurance are given in the participants information sheet.

Participants will be insured with a national insurance partner. The name and contact details for the insurer will be provided on the informed consent form.

f. Regulatory Requirements and GCP

The investigators at all sites are responsible for and should warrant that the conduct of the study shall be compliant to ICH-GCP, local regulatory requirements with the EC-approved research protocol.

The investigators will ensure, that this study is conducted in full conformance with the principles of the "Declaration of Helsinki" (as amended at the 64th WMA General Assembly, Fortaleza, Brazil, October 2013) and with the national laws and regulations of the country in which the clinical trial is conducted.

g. Research Ethics

Patients who will participate on this clinical trial will receive the third SARS-CoV-2 vaccination in a controlled way and will be informed about their antibody results immediately. Knowledge gained from this study may be helpful to determine efficacy of additional SARS-CoV-2 vaccination. Additional risk equates to the risk of blood draw in general. Therefore pain, local irritation, small bruises, local damage of nerves but also dizziness and infections can occur. Assessed data will be stored from the principal

investigator. Only authorized team members have access to the data. Processing of the data is only performed on password protected computers at the General Hospital Vienna.

Study Organization

h. Data Collection and Case Report From

For each subject enrolled, regardless of the study drug initiation, an CRF must be completed by the investigator or a designated sub-investigator. This also applies to those subjects, who fail to complete the study. If a subject withdraws from the study, the reason must be noted on the CRF. Case report forms are to be completed on an ongoing basis. Entries and corrections will only be performed by study site staff, who have been authorized by the investigator. Entry errors have to be corrected according the ICH-GCP Guidelines.

The entries will be checked by trained personnel (monitor) and any errors or inconsistencies will be checked and corrected immediately.

Data, collected at all visits, are entered into an Excel sheet. The CRFs constitute source documents established before study onset as detailed in the monitoring plan. The Maintenance of the study database will be performed by the Division of Rheumatology of the Medical University of Vienna.

The investigator shall maintain the records of drug disposition and the final CRF´s for a minimum of 15 years after the study closure.

i. Monitoring

The principal investigator will ensure, that the trial will be appropriately monitored by ensuring that all the rights of the subject are adequately protected, that the trial data are accurate, complete and verifiable from source documents and that the conduct of the trial is in compliance with the protocol and its subsequent amendments, with GCP and with applicable regulatory requirements. Risk-adapted monitoring will be performed by a contract research organization.

The sponsor investigator will ensure, that monitoring activities occur following a pre-defined monitoring plan. Monitoring of the trial will be performed by CW-Research & Management GmbH, Auhofstraße 84/3/39, A-1130 Vienna.

The investigators will verify, that for all patients a written informed consent is obtained before each subject´s participation in the trial. The investigators will also ensure, that all patients enrolled will be eligible according to the in- and exclusion criteria as defined in the protocol.

The on-site monitors will be responsible for verifying, that the appropriate assurances and certification for training in the protection of human subjects are in place at the site prior to the initiation of the protocol. They will provide education to all site staff regarding the conduct of the study according to good clinical practices (GCP´s). The sponsor will be responsible for ensuring, that informed consent has been obtained for each patient. These records will be verified during the monitoring/auditing visits that will be performed by the designated monitor.

Monitoring visits are planned at the beginning, during and at the end of the study according to the monitoring plan.

j. Audit and inspections

All investigators agree to accept audits and inspections by the competent authorities during and after completion of the study. All data and documents may be subject to audits and regulatory inspection.

k. Relevant protocol deviations

All protocol deviations will be listed in the study report and assessed as to their influence on the quality of the study analysis. No deviations from the protocol and of any type will be made without complying with all IRB/EC established procedures in accordance with applicable regulations.

l. Provision and handling for study medication

Vaccines for the study will be provided by the general Vaccination Board of the County of Vienna. Patients will be vaccinated utilizing the Vienna General Hospital Vaccination infrastructure (“Impfstrasse”, 4Süd). Study vaccines will be stored as recommended by the manufacturer’s instructions and prepared at the date of vaccination by the institutional pharmacy of the general hospital of Vienna.

m. Accountability for study medication

The investigator or his/her representative will verify, that study drug supplies are received intact and in the correct amounts. This will be documented with sign and date, as well as the arrival of drugs. All sheets will be kept in the site files as a record of what was received. Additionally, an investigational product accountability log will be documented, including, but not limited to, date received, lot number, kit number, date dispensed, subject number and the identification of the person dispensing the drug.

9. STATISTICAL ANALYSIS

9.1 Sample size considerations

Part A) According to the available number of patients under immunosuppressive therapy, including estimates of non-responders to a standard protocol of mRNA vaccination [10, 11] and expected participation rates, we will attempt to include 150 patients into this trial. Based on a Chi² test comparing 3rd mRNA versus vector vaccine, this number of patients will allow to achieve at least 80% power at a minimal detectable difference of 21% (10% of responders in the mRNA vaccine group versus 31% of responders in the vector vaccine group) with 57 patients per group.

For differences smaller than the minimum detectable difference statistical significance will not be possible to claim.

Part B) The estimated response to a second boost defined as achievement of > 1.500 U/ml of SARS-CoV-2 antibody titre will be 70%. To test that immunocompromised patients will have a similar rate of response after a second boost of mRNA vaccination compared to healthy controls we plan to include a total of 65 patients and 65 healthy controls. We attempt to recruit 75 per group allowing 15% of drop-outs. This number will allow to achieve at least 80% power based on non-inferiority (allowing a non-inferiority limit of a maximum of 20%).

9.2 Relevant protocol deviations

All protocol deviations will be listed in the study report. Major deviations regarding subjects' safety will lead to withdrawal.

9.3 Endpoints analysis

For Part A, the primary endpoint is defined as difference in SARS-CoV-2 antibody seroconversion rate by week 4 after the third SARS-CoV-2 vaccination between the two vaccine groups (either mRNA vaccine or vector vaccine). Patients who developed antibodies against SARS-CoV-2 will be defined as responders and patients without detectable antibodies will be classified as non responders. The difference between both groups will be analyzed using Chi-squared test.

For Part B) the primary endpoint is specified as comparisons of rate of response (antibody titres >1.500 U/ml) to a second boost vaccination between immunocompromised patients and healthy controls. The difference between both groups will be analyzed using Chi-squared test.

The secondary endpoints (see section 7.3.2) will be analyzed as following:

Comparisons of absolute and relative change in vaccine titers and delta of T-cell response before and after vaccination will be compared by a non-paired T-test or Mann Whitney U test, depending on the distribution of the data between 1) patients receiving different vaccine groups (either mRNA vaccine or vector vaccine; Part A), 2) patients with or without B-cells (Part A) or 3) immunocompromised patients and healthy controls (Part B).

In Part A, factors associated with seroconversion status (such as age, type of immunosuppressive therapy (including prednisone) and type of vaccine) will be analyzed by univariate and multivariate logistic regression models.

In Part B correlation of antibody titres before and after second boost will be calculated using Spearman correlation for the total group as well as separately for immunocompromised patients and healthy controls. Factors associated with total antibody titre as well as absolute and relative changes of antibody titre before and after second boost vaccination will be tested in univariate analyses (Spearman correlation for continuous variables such as age, steroid dose, time between the vaccination; ANOVA or Wilcoxon test for categorical variables such as type of immunosuppressive agent, disease entity). Multivariate analyses will be performed with multivariate regression analyses. Further secondary endpoints as well as safety will be investigated by using descriptive statistics (frequencies, tendencies and variation).

For the proposed study an intention-to-treat (IIT) analysis will be performed.

9.4 Missing, unused and spurious data

Only subjects for whom data are available will be included in the statistical analysis. Missing values will neither be replaced nor estimated.

9.5 Interim analysis

Part A and Part B will be analysed separately.

9.6 Software program(s)

Statistical analysis will be performed using R software, GraphPad Prism or SPSS Statistics (Version 17.0 or higher).

10. DISCLOSURE OF DATA

The investigator will publish the results of the study as an original scientific report in a peer reviewed scientific journal. In addition, the findings will be presented as oral and poster presentations at international scientific meetings. All obtained data and information will be regarded confidentially.

No data, results or any information of this study may be used for publication without prior agreement of the principal investigator, who is mentioned on the title page of this protocol. The authors of a publication, manuscript, article, abstract or oral presentation, are those, who contribute to the results of the study and/or the writing of the paper. The principal investigator will write the first draft of the paper and – if applicable – delivers the related presentations. He will also be the first author of the main efficacy manuscript. The correspondence address mentioned in each publication is the Sponsor-investigator´s address, as specified on the front-page. Additional co-authors will be determined based on their contributions during the course of the trial.

11. REFERENCES

1. Anderson EJ, Rouphael NG, Widge AT, Jackson LA, Roberts PC, Makhene M, et al. Safety and Immunogenicity of SARS-CoV-2 mRNA-1273 Vaccine in Older Adults. N Engl J Med. 2020; 383(25): 2427-38.
2. Polack FP, Thomas SJ, Kitchin N, Absalon J, Gurtman A, Lockhart S, et al. Safety and Efficacy of the BNT162b2 mRNA Covid-19 Vaccine. New England Journal of Medicine. 2020; 383(27): 2603-15.
3. Walsh EE, Frenck RW, Jr., Falsey AR, Kitchin N, Absalon J, Gurtman A, et al. Safety and Immunogenicity of Two RNA-Based Covid-19 Vaccine Candidates. N Engl J Med. 2020; 383(25): 2439-50.
4. Mulligan MJ, Lyke KE, Kitchin N, Absalon J, Gurtman A, Lockhart S, et al. Phase I/II study of COVID-19 RNA vaccine BNT162b1 in adults. Nature. 2020; 586(7830): 589-93.
5. Sahin U, Muik A, Derhovanessian E, Vogler I, Kranz LM, Vormehr M, et al. COVID-19 vaccine BNT162b1 elicits human antibody and T(H)1 T cell responses. Nature. 2020; 586(7830): 594-9.
6. Voysey, M., et al., Safety and efficacy of the ChAdOx1 nCoV-19 vaccine (AZD1222) against SARS-CoV-2: an interim analysis of four randomised controlled trials in Brazil, South Africa, and the UK. Lancet, 2021. 397(10269): p. 99-111.
7. D'Silva KM, Serling-Boyd N, Wallwork R, Hsu T, Fu X, Gravallese EM, et al. Clinical characteristics and outcomes of patients with coronavirus disease 2019 (COVID-19) and rheumatic disease: a comparative cohort study from a US 'hot spot'. Ann Rheum Dis. 2020; 79(9): 1156-62.
8. Simon D, Tascilar K, Fagni F, et al. SARS-CoV-2 vaccination responses in untreated, conventionally treated and anticytokine-treated patients with immune-mediated inflammatory diseases. Ann Rheum Dis. 2021;annrheumdis-2021-220461.
9. Emary, K.R.W., et al., Efficacy of ChAdOx1 nCoV-19 (AZD1222) vaccine against SARS-CoV-2 variant of concern 202012/01 (B.1.1.7): an exploratory analysis of a randomised controlled trial. Lancet, 2021. 397(10282): p. 1351-1362.
10. Rozen-Zvi B, Yahav D, Agur T et al. Antibody response to SARS-CoV-2 mRNA vaccine among kidney transplant recipients: a prospective cohort study. Clinical Microbiology and Infection,2021, ISSN 1198-743X, https://doi.org/10.1016/j.cmi.2021.04.028.
11. Ciotti JR, Valtcheva MV, Cross AH. Effects of MS disease-modifying therapies on responses to vaccinations: A review. Mult Scler Relat Disord. 2020 Oct;45:102439.
