# Supplementary material for: Heterologous vector versus homologous mRNA COVID-19 booster vaccination in non-seroconverted immunosuppressed patients: a randomized controlled trial
Source: Nat Commun. 2022 Sep 12;13:5362. doi: 10.1038/s41467-022-33036-y (PMC9467419; doi:10.1038/s41467-022-33036-y)
Supplement: Supplementary file 2 — Reporting Summary [file 41467_2022_33036_MOESM2_ESM.pdf]

nature portfolio

Corresponding author(s): Leonhard X. Heinz, Michael Bonelli

Last updated by author(s): Aug 20, 2022

Reporting Summary

Nature Portfolio wishes to improve the reproducibility of the work that we publish. This form provides structure for consistency and transparency in reporting. For further information on Nature Portfolio policies, see our Editorial Policies and the Editorial Policy Checklist.

Statistics

For all statistical analyses, confirm that the following items are present in the figure legend, table legend, main text, or Methods section.

n/a	Confirmed	
☐	☒	The exact sample size (*n*) for each experimental group/condition, given as a discrete number and unit of measurement
☐	☒	A statement on whether measurements were taken from distinct samples or whether the same sample was measured repeatedly
☐	☒	The statistical test(s) used AND whether they are one- or two-sided<br>*Only common tests should be described solely by name; describe more complex techniques in the Methods section.*
☐	☒	A description of all covariates tested
☒	☐	A description of any assumptions or corrections, such as tests of normality and adjustment for multiple comparisons
☐	☒	A full description of the statistical parameters including central tendency (e.g. means) or other basic estimates (e.g. regression coefficient) AND variation (e.g. standard deviation) or associated estimates of uncertainty (e.g. confidence intervals)
☐	☒	For null hypothesis testing, the test statistic (e.g. *F*, *t*, *r*) with confidence intervals, effect sizes, degrees of freedom and *P* value noted<br>*Give P values as exact values whenever suitable.*
☒	☐	For Bayesian analysis, information on the choice of priors and Markov chain Monte Carlo settings
☒	☐	For hierarchical and complex designs, identification of the appropriate level for tests and full reporting of outcomes
☒	☐	Estimates of effect sizes (e.g. Cohen's *d*, Pearson's *r*), indicating how they were calculated

Our web collection on statistics for biologists contains articles on many of the points above.

Software and code

Policy information about availability of computer code

Data collection	Flow cytometry data were acquired on a FACS Canto II flow cytometer using FACS Diva 8.0 software (Becton Dickinson). ELISpot data were collected using a Bioreader 5000 Pro-S/BR177 and Bioreader software generation 10 (Bio-Sys). Microsoft Excel 365 was used for participant data collection.
Data analysis	FACS Diva 8.0 software (Becton Dickinson) was used for analysis of flow cytometry data. HLA-specific antibody testing was analyzed using HLA Fusion software V4.2 (OneLambda) GraphPad Prism (version 9.1.0) and “R” version 4.0.3 (R Development Core Team. Vienna, Austria) were used for graphical presentation and statistical analysis. Following packages were utilized: “ggplot2” (V3.3.2), “ggbeeswarm” (V0.6.0), “corrplot” (V0.88) and “sjPlot” (V2.8.9) for creating plots, “pwr” (V1.3) for power calculation and “tableone” (V0.12.0) to create baseline tables.

For manuscripts utilizing custom algorithms or software that are central to the research but not yet described in published literature, software must be made available to editors and reviewers. We strongly encourage code deposition in a community repository (e.g. GitHub). See the Nature Portfolio guidelines for submitting code & software for further information.

Data

Policy information about availability of data

All manuscripts must include a data availability statement. This statement should provide the following information, where applicable:

- Accession codes, unique identifiers, or web links for publicly available datasets
- A description of any restrictions on data availability
- For clinical datasets or third party data, please ensure that the statement adheres to our policy

Source data of the main figures are provided as a source data file and in supplementary table 5. De-identified participant data is available upon request. Proposals must be submitted to the corresponding authors and will be reviewed within two months. Once the proposal has been approved, data can be transferred through a secure online platform after the signing of a data access agreement and a confidentiality agreement.

Human research participants

Policy information about studies involving human research participants and Sex and Gender in Research.

Reporting on sex and gender	The findings in this trial apply to both sex and gender. Neither sex or gender were considered in the study design. Sex was assigned as reported by the Austrian national security database. Gender was not assessed in this trial. Sex is displayed in Table 1 in the trial data. No sex and gender based analyses were performed.
Population characteristics	Adult (age ≥ 18 years) patients under immunosuppressive treatment without measurable SARS-CoV-2 spike protein-specific antibodies at least four weeks after their second COVID-19 vaccination were eligable. Most important inclusion criteria was previous vaccination with two doses of an mRNA vaccine (BNT162b2 or mRNA-1273). Major exclusion criteria were known allergies to vaccines, previous infection with SARS-CoV-2, detectable anti-spike antibodies at the time of inclusion or prior use of B-cell-depleting agents, such as rituximab.
Recruitment	Patients were recruited at the Vienna General Hospital. Patients without detectable SARS-CoV-2 spike protein-specific antibodies antibodies were referred by their treating physician to the trial staff. All patients had to fulfill inclusion critera, and main trail outcomes were all determined by laboratory assessment in a blinded manner, thus minimizing self selection bias. Patients were stratified according whether they had detectable or non-detectable peripheral B-cell count, minimzing the risk of data skewing by this influential factor. Furthermore, patients were blinded to the intervention to assess the adverse events independentily of the vaccine applied.
Ethics oversight	Ethics comitee of the Medical University of Vienna, Austria

Note that full information on the approval of the study protocol must also be provided in the manuscript.

Field-specific reporting

Please select the one below that is the best fit for your research. If you are not sure, read the appropriate sections before making your selection.

☒ Life sciences ☐ Behavioural & social sciences ☐ Ecological, evolutionary & environmental sciences

For a reference copy of the document with all sections, see nature.com/documents/nr-reporting-summary-flat.pdf

Life sciences study design

All studies must disclose on these points even when the disclosure is negative.

Sample size	The study sample size was targeted at 150 individuals, thus based on a Chi-squared test comparing the two groups, the minimal detectable difference was 21% at a power of 80%. During recruitment, routine access to the additional vaccination dose, as offered in our trial, was facilitated for high-risk patients in Austria, which substantially slowed our inclusion rates. To allow timely study completion, the decision was taken to stop inclusion prematurely at 75 enrolled patients, which would provide power to detect an effect size of at least 32% for our patient cohort. The observed effect size was ultimately larger, which eliminates considerations about a potential type II error, i.e. failure to reject the null hypothesis although it is actually false.
Data exclusions	All patients who were vaccinated completed the trial and were analyzed (see study flow diagram - Figure 1). As established previously, a total of 1 ×10e5 cells per well were incubated with PHA to obtain a spot frequency of at least 60 spots per well, corresponding to 600 spots/10e6 cells. In ELISpot assays, samples with a PHA response < 600 SFC/10e6 PBMCs were excluded.
Replication	All measurements were performed in multiple biological replicates (n as indicated in the Figure Legends). In addition, cellular immune responses by ELISpot have been performed in technical duplicates, no samples were exluded for technical reasons.
Randomization	Patients were block-randomized in a 1:1 ratio based on the presence or absence of peripheral B-cells using a computerized algorithm (Randomizer).

Blinding	Laboratory assessors and patients were blinded to the type of vaccine used. Blinding of vaccines was ensured by the Central Pharmacy of the Vienna General Hospital, where dose aliquots were pre-arranged in syringes without reference to the vaccine type used. Laboratory assessors had no information on vaccine type used in each individual patient.

Reporting for specific materials, systems and methods

We require information from authors about some types of materials, experimental systems and methods used in many studies. Here, indicate whether each material, system or method listed is relevant to your study. If you are not sure if a list item applies to your research, read the appropriate section before selecting a response.

Materials & experimental systems

n/a	Involved in the study	
☐	☒	Antibodies
☒	☐	Eukaryotic cell lines
☒	☐	Palaeontology and archaeology
☒	☐	Animals and other organisms
☐	☒	Clinical data
☒	☐	Dual use research of concern

Methods

n/a	Involved in the study	
☒	☐	ChIP-seq
☐	☒	Flow cytometry
☒	☐	MRI-based neuroimaging

Antibodies

Antibodies used

Quantification of memory B cells by flow cytometry:

Reagents / Lot Nr. / Source / Identifier / Dilution
Mouse anti-human CD19 FITC (clone HIB19) / 6098580 / BD Bioscience / Cat# 555412 / 1:20
Mouse anti-human CD27 PE (clone L128) / 0342780 / BD Bioscience / Cat# 340425 / 1:20
Mouse anti-human CD38 PERCP Cy 5.5 (clone HIT2) / 1104567 / BD Bioscience / Cat# 551400 / 1:100
Mouse anti-human IgD PE Cy7 (clone IA6-2) / 5313841 / BD Bioscience / Cat# 561314 / 1:100
Fixable Viability Dye eFluor-506 / 2095423 / Thermo Fisher Scientific / Cat# 65-0866 / 1:666

Quantification of peripheral leukocytes (all antibodies provided by Becton-Dickinson):

Antibody / Catalogue number / Clone / LOT-number / Dilution
CD4-PerCP-cy5.5 / 332772 / SK3 (Leu-3a) / 1294296 / 1:20
CD8-APC-Cy7 / 348813 / SK1 (Leu-2a) / 2045876 / 1:40
CD19-PE-Cy7 / 341113 / SJ25C1 / 2060995 / 1:40
BD Simultest CD3-FITC, CD16 + CD56-PE / 342403 / SK7, B73.1, MY3 / 1089816 / 1:20
CD14-APC / 345787 / MFP9 / 1314052 / 1:40
HLA-DR-V450 / 655874 / L243 / 2039201 / 1:40
CD45-V500 / 655873 / 2D1 (HLe-1) / 2040526 / 1:40

Interferon-gamma-specific ELISpot Assay (all antibodies from Mabtech):

Coating: Anti-human IFN-γ mAb (1-D1K), product code: 3420-3-1000 / 1:67
Detection: Anti-human IFN-γ mAb (7-B6-1), biotin, product code: 3420-6-1000 / 1:1000

Validation

All antibodies were bought from commercial vendors and validation for indicated species and applications are provided on the manufacturers websites as follows:

Memory B cells:
CD19 FITC: https://www.bdbiosciences.com/en-at/products/reagents/flow-cytometry-reagents/research-reagents/single-color-antibodies-ruo/fitc-mouse-anti-human-cd19.555412
CD27 PE: https://www.bdbiosciences.com/en-at/products/reagents/flow-cytometry-reagents/clinical-discovery-research/single-color-antibodies-ruo-gmp/pe-mouse-anti-human-cd27.340425
CD38 PERP Cy5.5: https://www.bdbiosciences.com/en-at/products/reagents/flow-cytometry-reagents/research-reagents/single-color-antibodies-ruo/percp-cy-5-5-mouse-anti-human-cd38.551400
IgD PE Cy7: https://www.bdbiosciences.com/en-at/products/reagents/flow-cytometry-reagents/research-reagents/single-color-antibodies-ruo/pe-cy-7-mouse-anti-human-igd.561314
Fixable Viability Dye: https://www.thermofisher.com/order/catalog/product/65-0866-14

Peripheral leucocytes:
CD4-PerCP-cy5.5: https://www.bdbiosciences.com/en-at/products/reagents/flow-cytometry-reagents/clinical-diagnostics/single-color-antibodies-asr-ivd-ce-ivd/cd4-percp-cy-5-5.332772
CD8-APC-Cy7: https://www.bdbiosciences.com/en-at/products/reagents/flow-cytometry-reagents/clinical-diagnostics/single-color-

antibodies-asr-ivd-ce-ivd/cd8-apc-cy-7.348813
CD19-PE-Cy7: https://www.bdbiosciences.com/en-at/products/reagents/flow-cytometry-reagents/clinical-diagnostics/single-color-antibodies-asr-ivd-ce-ivd/cd19-pe-cy-7.341113
CD3-FITC, CD16 + CD56-PE: https://www.bdbiosciences.com/en-at/products/reagents/flow-cytometry-reagents/clinical-diagnostics/multicolor-cocktails-and-kits-ivd-ce-ivds/cd3-fitc-cd16-cd56-pe.342403
CD14-APC: https://www.bdbiosciences.com/en-at/products/reagents/flow-cytometry-reagents/clinical-diagnostics/single-color-antibodies-asr-ivd-ce-ivd/cd14-apc.345787
HLA-DR-V450: https://www.bdbiosciences.com/en-at/products/reagents/flow-cytometry-reagents/clinical-diagnostics/single-color-antibodies-asr-ivd-ce-ivd/anti-hla-dr-v450.655874
CD45-V500: https://www.bdbiosciences.com/en-at/products/reagents/flow-cytometry-reagents/clinical-diagnostics/single-color-antibodies-asr-ivd-ce-ivd/cd45-v500-c.655873

ELIspot:
Coating: https://www.mabtech.com/products/anti-human-ifn-gamma-antibody-1-d1k-unconjugated-3420-3#tabs-min-2
Detection: https://www.mabtech.com/products/anti-human-ifn-gamma-antibody-7-b6-1-biotin-3420-6#tabs-min-2

Clinical data

Policy information about clinical studies
All manuscripts should comply with the ICMJEguidelines for publication of clinical research and a completedCONSORT checklist must be included with all submissions.

Clinical trial registration: EudraCT No.: 2021-002693-10

Study protocol: The detailed study protocol is included in the supplementary information.

Data collection: All trial visits were conducted monocentric in a tertiary hospital (Vienna General Hospital). The trial started on July 22nd, 2021, with inclusion of the first patient. The last patient finalized the 4-week follow-up on October 8th, 2021.

Outcomes: The primary outcome of the study was the difference in antibody seroconversion rates between the two intervention groups (vector versus mRNA vaccine). According to manufacturer’s specification, seroconversion was defined as an anti-RBD antibody concentration of over >0.8 BAU/ml. Secondary endpoints included overall seroconversion rate and SARS-CoV-2 antibody levels at week four and cellular immune response before and one week following vaccination, furthermore the assessment of safety included incidence and severity of adverse events over 28 days.

Flow Cytometry

Plots

Confirm that:

- [x] The axis labels state the marker and fluorochrome used (e.g. CD4-FITC).
- [x] The axis scales are clearly visible. Include numbers along axes only for bottom left plot of group (a 'group' is an analysis of identical markers).
- [x] All plots are contour plots with outliers or pseudocolor plots.
- [x] A numerical value for number of cells or percentage (with statistics) is provided.

Methodology

Sample preparation:

Determination of SARS-CoV-2 spike-specific memory B-cells:
For detection of SARS-CoV-2 Spike (S) protein-specific memory B-cells, biotinylated S protein antigen was individually mixed with streptavidin-APC and streptavidin-BV421 probes. Streptavidin-APC-Cy7 without biotinylated protein was used as a decoy probe to gate out B cells that non-specifically bind streptavidin. The S protein antigen probes and decoy probe were mixed in Brilliant Buffer (BD Bioscience, Cat# 566349) containing 5µM free D-biotin (to minimize potential cross-reactivity between probes) and for staining 3x106 cryopreserved PBMC prepared in 96-well U-bottom plates were incubated with 50µL antigen probe cocktail, containing 100ng S protein per probe, at 4°C for one hour. Thereafter, surface staining was performed with directly-labeled monoclonal Abs towards human CD19 (FITC, clone HIB19), human CD27 (PE, clone L128), human CD38 (PerCP-Cy5.5, clone HIT2) and human immunoglobulin D (IgD) (PE-Cy7, clone IA6-2), all BD Bioscience, in Brilliant Buffer at 4°C for 30 min. Dead cells were excluded by using Fixable viability dye eFluor-506 (eBioscience, now Thermo Fisher Scientific). Data were acquired on a FACS Canto II flow cytometer by gating on cells with forward/side light scatter properties of lymphocytes and analyzed with FACS Diva 8.0 software. S-specific memory B-cells were quantified as percentages of total memory B-cells, including the CD19+ un-switched (IgD+/CD27+), switched (IgD-/CD27+) and double-negative (IgD-/CD27-) memory subset.

Quantification of peripheral leukocytes:
Flow cytometry (FACSCanto II, Becton Dickinson, San Jose, California, USA) was utilized to determine the phenotype of peripheral leukocytes. Hereby staining of whole blood followed by lyse-wash procedure (Becton Dickinson) was performed. The following monoclonal antibodies were used to classify lymphocytes subpopulations (all provided by Becton Dickinson): fluorescein isothiocyanate (FITC)-labelled anti-CD3, phycoerythrin (PE)-labelled anti-CD16+56+, peridinin-chlorophyll-protein (PerCP)-cy5.5-labelled anti-CD4, PE-Cy7-labelled anti-CD19, allophycocyanin (APC)-Cy7-labelled anti-CD8, V450-labelled anti-human leukocyte antigen (HLA)-DR, V500-labelled anti-CD45 and APC-labelled anti-CD14. Results are presented either as absolute numbers or as percentage of a subpopulation among total lymphocytes.

Instrument	FACS Canto II flow cytometer, Becton Dickinson, San Jose, California, USA
Software	FACS Diva 8.0 software
Cell population abundance	No cell sorting was used in this study.
Gating strategy	FSC/SSC was used to select lymphocytes and remove doublets. Dead cells were excluded by using fixable viability dye eFluor-506. CD19+ B-cells were gated and S-specific memory B-cells were quantified as percentages of total memory B-cells, including the CD19+ un-switched (IgD+/CD27+), switched (IgD-/CD27+) and double-negative (IgD-/CD27-) memory subset after exclusion of cells positive for streptavidin-APC-Cy7 without biotinylated protein that was used as a decoy probe to gate out B cells that unspecifically bind streptavidin.

☒ Tick this box to confirm that a figure exemplifying the gating strategy is provided in the Supplementary Information.
